# Mandibulofacial dysostosis with alopecia results from ET_A_R gain-of-function mutations via allosteric effects on ligand binding

**DOI:** 10.1172/JCI151536

**Published:** 2023-02-15

**Authors:** Yukiko Kurihara, Toru Ekimoto, Christopher T. Gordon, Yasunobu Uchijima, Ryo Sugiyama, Taro Kitazawa, Akiyasu Iwase, Risa Kotani, Rieko Asai, Véronique Pingault, Mitsunori Ikeguchi, Jeanne Amiel, Hiroki Kurihara

**Affiliations:** 1 Department of Physiological Chemistry and Metabolism, Graduate School of Medicine, The University of Tokyo, Tokyo, Japan.; 2 Graduate School of Medical Life Science, Yokohama City University, Yokohama, Japan.; 3 INSERM UMR 1163, Institut Imagine and Université Paris-Cité, Paris, France.; 4 Department of Medical Science, Graduate School of Medicine, University of Hiroshima, Hiroshima, Japan.; 5 Department of Genomic Medicine for Rare Diseases, Hôpital Necker-Enfants Malades, AP-HP, Paris, France.; 6 Center for Computational Science, RIKEN, Yokohama, Japan.

**Keywords:** Development, Genetics, G protein&ndash;coupled receptors, Mouse models, Structural biology

## Abstract

Mutations of G protein–coupled receptors (GPCRs) cause various human diseases, but the mechanistic details are limited. Here, we establish p.E303K in the gene encoding the endothelin receptor type A (ET_A_R/*EDNRA*) as a recurrent mutation causing mandibulofacial dysostosis with alopecia (MFDA), with craniofacial changes similar to those caused by p.Y129F. Mouse models carrying either of these missense mutations exhibited a partial maxillary-to-mandibular transformation, which was rescued by deleting the ligand endothelin 3 (ET3/*EDN3*). Pharmacological experiments confirmed the causative ET_A_R mutations as gain of function, dependent on ET3. To elucidate how an amino acid substitution far from the ligand binding site can increase ligand affinity, we used molecular dynamics (MD) simulations. E303 is located at the intracellular end of transmembrane domain 6, and its replacement by a lysine increased flexibility of this portion of the helix, thus favoring G protein binding and leading to G protein–mediated enhancement of agonist affinity. The Y129F mutation located under the ligand binding pocket reduced the sodium-water network, thereby affecting the extracellular portion of helices in favor of ET3 binding. These findings provide insight into the pathogenesis of MFDA and into allosteric mechanisms regulating GPCR function, which may provide the basis for drug design targeting GPCRs.

## Graphical abstract

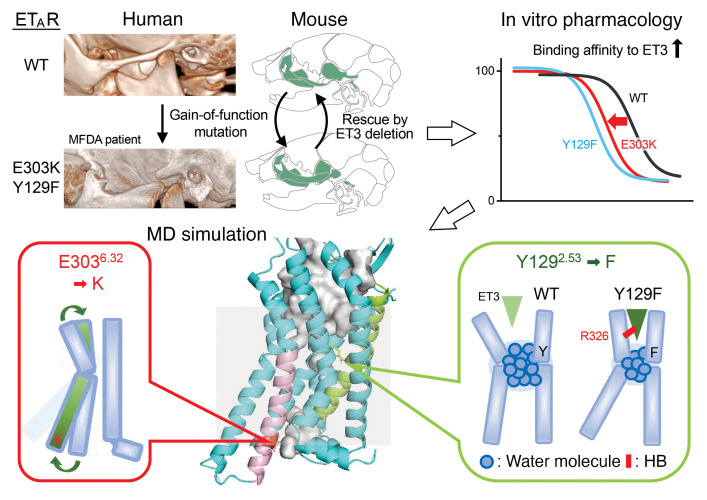

## Introduction

G protein–coupled receptor–mediated (GPCR-mediated) signal transduction plays essential roles in various pathophysiological and physiological events. Recent progress in structural biology has accelerated the mechanistic understanding of GPCR activation. In particular, full agonist binding to the orthosteric binding site activates heterotrimeric G proteins through outward movement of the sixth transmembrane helix (TM) on the receptor’s intracellular side (1–5). Conversely, G protein–mediated enhancement of agonist affinity has also been suggested as a mode of allosteric regulation (6, 7). Thus, conformational changes on both sides of the membrane are closely linked and mutually affect receptor functions. In addition, the Na^+^/water pocket has emerged as a key structure, common to class A GPCRs such as β_2_AR, opioid δ-OR, PAR1, adenosine A_2_R, and BLT1 (8, 9). It maintains the inactive state by forming a polar interaction between Na^+^ and D^2.50^ — where the superscript refers to the Ballesteros-Weinstein numbering system (10) — and a hydrogen bonded network of surrounding water molecules and hydrophilic residues. The collapse of this pocket by the release of Na^+^ and water molecules can cause a conformational change, allosterically affecting the ligand-binding pocket and receptor activation (8, 11).

Endothelin (ET/*EDN*) receptors are members of the class A GPCR family and consist of 2 isoforms, type A (ET_A_R/*EDNRA*) and type B (ET_B_R/*EDNRB*), with different affinities to 3 ligands ET1, ET2, and ET3. ET_A_R binds to ET1 and ET2 with much higher affinity than to ET3, whereas ET_B_R nonselectively binds all 3 ligands (12, 13). The role of ET signaling has been extensively studied in the cardiovascular system. ET receptor antagonists are now in clinical use for pulmonary arterial hypertension and systemic sclerosis, and are being considered for intractable hypertension, heart failure, kidney diseases, and cancers, for which the development of different types of ET receptor antagonists may be necessary (14).

ET signaling is also critical for development of neural crest cells (NCCs) during embryogenesis (15–19). In craniofacial development, ET1 is secreted from the epithelium and core mesoderm in the ventral domain of the pharyngeal arches (PAs) to act on ET_A_R-expressing migrating cranial NCCs (20, 21). In mice, inactivation of ET1-ET_A_R signaling causes a loss of lower jaw identity and a transformation into a mirror-image duplication of the upper jaw (15, 22, 23). Conversely, ectopic activation of ET_A_R induced by the forced expression of ET1 in the maxillary prominence results in upper-to-lower jaw transformation in mice (24, 25). In humans, loss-of-function mutations in *EDN1* or dominant negative mutations in *PLCB4* and *GNAI3* — predicted to act downstream of ET_A_R — cause auriculocondylar syndrome (ACS), characterized by a “question mark” shape of the external ears and a lower jaw that resembles an upper jaw (26–30). Also, a biallelic loss-of-function variant in *EDNRA* has been reported in a patient with severe hypoplasia of the lower jaw, anotia, and cardiac defects (oro-oto-cardiac syndrome; ref. 31).

Recently, 2 missense mutations of *EDNRA*, p.Y129^2.53^F and p.E303^6.32^K, were identified as the cause of mandibulofacial dysostosis with alopecia (MFDA, MIM 616367) (32). Patients with the p.Y129F mutation have no temporomandibular joints, flattened condyles, and a thickened malar bone, highly reminiscent of mice with ectopic ET_A_R activation in the upper jaw (24) and mice deficient in *Six1*, a negative regulator of *Edn1* (33). Together with previous findings demonstrating increased affinity of the p.Y129F mutant ET_A_R for ET3 in vitro (34), a gain-of-function mechanism was proposed for MFDA caused by the p.Y129F mutation. However, no craniofacial imaging was available for the single published patient harboring p.E303K (case 4 in ref. 32), and the consequences of this mutation have remained unclear.

Here, we report a further patient with MFDA patient carrying the p.E303K *EDNRA* mutation with detailed clinical characterization, and knockin mouse models for both p.Y129F and p.E303K. Phenotypic analyses and pharmacological studies demonstrated gain-of-function effects that are largely dependent on enhanced ET3 binding affinity, although via different mechanisms for each mutation. Molecular dynamics (MD) simulations provided a structural basis for the functional changes of the mutant receptors, with potential relevance for the development of drugs targeting the ET receptor family.

## Results

### MFDA in a patient with the EDNRA mutation p.E303K.

**Figure 1:**
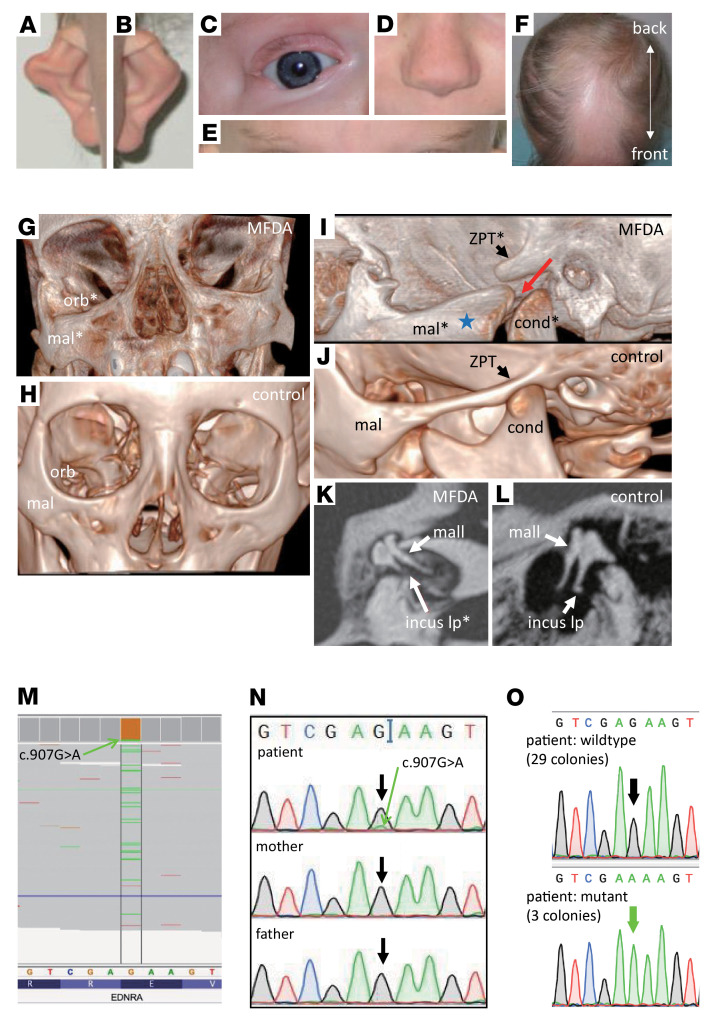
A patient with MFDA harboring the *EDNRA* mutation p.E303K, that occurred postzygotically. (**A–F**) Dysmorphic facial features and alopecia of the patient (see text for details). (**G–J**) High resolution craniofacial 3D CT-scans of the patient with MFDA (**G** and **I**) and an age-matched control (**H** and **J**). (**G** and **H**) Frontal views show hypertrophic, dysplastic, and asymmetric malar bones and an abnormal orbital floor in the patient (**G**) compared with the control (**H**). (**I** and **J**) Volume-rendering sagittal reconstructions of the temporomandibular articulations show a novel, tripartite temporo-mandibulo-malar articulation on the left side (red arrow) with increased malar bone volume (blue star) in the patient (**I**) compared with the control (**J**). (**K** and **L**) Oblique axial reconstructions of CT scans of the incus and malleus showing hypoplasia of the long process of the incus in the patient (**K**) compared with a control (**L**). (**M**) Next-generation sequencing reads showing mosaicism for the *EDNRA* variant c.907G>A (8%–11% of reads, in green) in the patient’s peripheral blood DNA. (**N**) Sanger sequencing of *EDNRA* amplified by PCR from leukocyte DNA of the patient and her parents, demonstrating low -evel mosaicism for the variant c.907G>A (green arrow) in the patient. (**O**) Sequencing of PCR products amplified from individual bacterial colonies, following transformation with cloned *EDNRA* PCR fragments from the patient, indicating 3/32 colonies (9 %) harboring the variant (green arrow) while 29 colonies harbored the WT allele (black arrow). Abbreviations: orb, orbital floor; mal, malar bone; cond, condyle of the mandible; ZPT, zygomatic process of the temporal bone; mall, malleus; incus lp, long process of the incus. An asterisk indicates structures that are dysmorphic compared with normal.

We investigated a girl presenting with symmetrical dysplastic ears (Figure 1, A and B); asymmetric lower eyelid coloboma that required surgery on the right side (Figure 1C); a squared nasal tip (Figure 1D); widely spaced, thin, horizontal eyebrows (Figure 1E); and sparse scalp hair with persistent frontal balding (Figure 1F). She has hearing aids for conductive hearing loss and generalized hypopigmentation compared with first degree relatives. Craniofacial 3D CT scans showed hypertrophic, dysplastic, asymmetric malar bones, abnormal orientation of the orbital floor, and an abnormal temporomandibular articulation on the left side (Figure 1, G–J). CT scans also showed abnormalities of the middle ear, including hypoplasia of the long process of the incus (Figure 1, K and L). The abnormal bones are all cranial NCC derivatives. Further detailed information of this patient is described in Supplemental Table 1; supplemental material available online with this article; https://doi.org/10.1172/JCI151536DS1 Next-generation sequencing revealed that the peripheral blood DNA of the patient was 8%–11% mosaic for the mutation c.907G>A; p.E303K in *EDNRA* (NM_001957.3) (Figure 1M). Low-level mosaicism of the mutation was confirmed by Sanger sequencing of a PCR product amplified from blood DNA (Figure 1N) and from PCR products amplified from bacterial colonies transformed with the cloned PCR product from the blood (Figure 1O). The variant was not detected in the mother or father (Figure 1N), indicating a postzygotic mutation. These results establish that p.E303K is a second-recurrent mutation causing MFDA, with structural changes of the jaw similar to those caused by p.Y129F.

### ET_A_R-Y129F and E303K mutations in mice recapitulate craniofacial manifestations of MFDA in humans.

**Figure 2:**
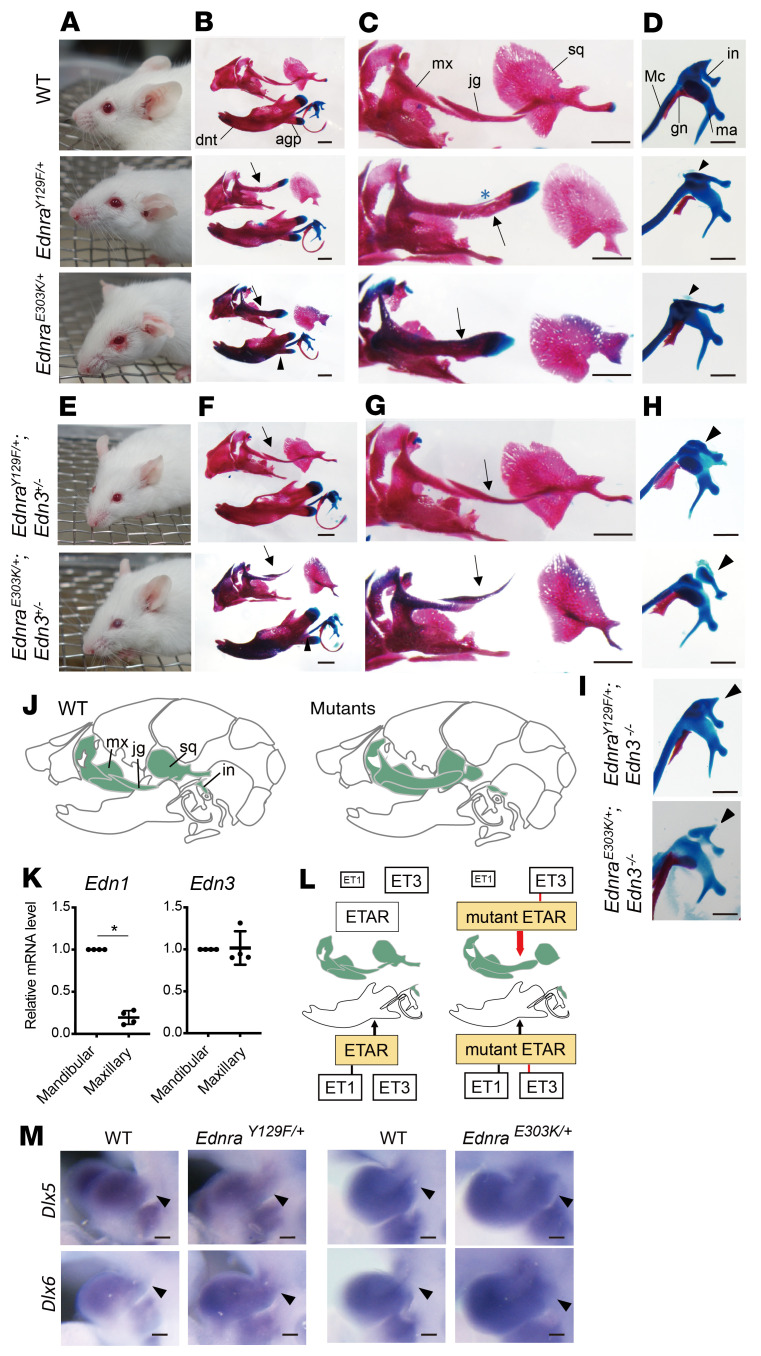
Craniofacial phenotypes of *Ednra* mutant mice are rescued by *Edn3* deletion. (**A–D**) Facial appearance and skeletal morphology of WT and heterozygous mutant (*Ednra^Y129F/+^*and *Ednra^E303K/+^*) mice. Mutant adult mice show over-folded pinnae and skin erosion around the eyelid (**A**). E18.5 bone and cartilage stains show transformation of the upper jaw components into a rod-shaped bone in the mutants (arrows in **B** and **C**) and deformity of the incus (arrowheads in **D**). The mandible is almost normal, except the angular process, which is slightly reduced (arrowhead in **B**). (**E**) Facial abnormalities in the *Ednra^Y129F/+^* and *Ednra^E303K/+^* mutants are variably rescued by heterozygous deletion of the *Edn3* gene. (**F** and **G**) Transformation of the upper jaw components in the mutants is rescued by heterozygous *Edn3* deletion (arrows). (**H**) deformity of the incus (arrowheads) is not rescued by heterozygous *Edn3* deletion. (**I**) The incus of the mutants is normalized by homozygous loss of *Edn3* (arrowheads). (**J**) Schematic diagrams indicating the skeletal components affected in the *Ednra* mutants (green). (**K**) Comparison of *Edn1* and *Edn3* mRNA levels at E9.5. 4 pairs of RNA samples were extracted from pools of mandibular or maxillary arches, in each of which 7 to 14 WT littermate samples were combined and were subjected to q-RT-PCR. *P < 0.05 by Mann-Whitney U test (*n* = 4). (**L**) Schematic diagrams summarizing the effect of ET signals in normal and pathogenic states. Red lines indicate ET3-dependent activation of mutant receptors. (**M**) *Dlx5* and *Dlx6* expression at E9.5 is restricted to the mandibular region of PA1 in WT embryos, whereas the expression of both genes extends into the maxillary region of PA1 in *Ednra^Y129F/+^*and *Ednra^E303K/+^* embryos (arrowheads). *n* = 3–6. agp, angular process; dnt, dentary; gn, gonial; in, incus; jg, jugal; ma, malleus; Mc, Meckel’s cartilage; mx, maxilla; sq, squamosal. Scale bars: 1 mm (**B**, **C**), 500 µm (**D**), and 100 µm (**M**)

The residues mutated in MFDA and their flanking sequences are highly conserved between humans and mice. Therefore, in order to investigate the developmental mechanism, we generated mouse models of MFDA by substituting Y129 or E303 with phenylalanine or lysine, respectively, using the CRISPR/Cas9 system. Mice carrying either heterozygous mutant allele (*Ednra^Y129F/+^* or *Ednra^E303K/+^*) showed over-folded pinnae, which was more severe for *Ednra^E303K/+^* than *Ednra^Y129F/+^*, and some mutant mice developed skin erosion around the eyelids, with sparse and coarse coat worsening with age (Figure 2A and Supplemental Figure 1A). Some *Ednra^Y129F/+^* and almost all *Ednra^E303K/+^* mice had open eyelids at birth (Table 1 and Supplemental Figure 1A). Skeletal analysis revealed similar abnormalities in NCC-derived maxillary components in both mutants. The zygomatic arch, composed of the jugal and zygomatic processes of the maxilla and squamosal, was deformed into a rod-shaped bone with a cartilaginous proximal end (Figure 2, B and C), on the top of which a vestigial jugal-like bone was also observed (Figure 2C blue asterisk). This rod-shaped bone appears similar to the dentary and condylar process, suggesting a partial transformation of the upper jaw toward mandibular identity. The incus was also deformed in the mutants with an appearance similar to the malleus (Figure 2D). The skeletal components affected in the mutants are illustrated in Figure 2J. No major abnormalities of the mandibular components were observed except for a slightly reduced angular process in *Ednra^E303K/+^* mice (Figure 2B arrowhead).

As previously described (24), ectopic activation of ET_A_R by forced ET1 expression resulted in the replacement of the ala temporalis with a Meckel’s-like cartilage, indicating a dorsal-to-ventral transformation of the first pharyngeal arch (PA1). *Ednra^Y129F/+^* and *Ednra^E303K/+^* embryos demonstrated intact formation of the ala temporalis (Supplemental Figure 2); however, the expression of *Dlx5* and *Dlx6*, homeobox transcription factors that act downstream of ET1-ET_A_R signaling to determine ventral identity in PA1, extended dorsally in mutant embryos (Figure 2M). These results suggest that the *Ednra* mutations had an ectopic gain-of-function effect in the dorsal PA1, although weaker than that observed upon forced expression of ET1.

The phenotype associated with either homozygous *Ednra* mutation was similar to that of the heterozygous mutants, except that the condylar process was reduced in *Ednra^E303K/E303K^* mice (Supplemental Figure 3C red arrow). Importantly, the homozygous mutations did not lead to a mandibular-to-maxillary transformation, which is a hallmark of inactivation of ET1-ET_A_R signaling in mice and of ACS in humans (Supplemental Figure 3, C and D, and Table 1).

### Deletion of Edn3 rescues the phenotype associated with Ednra^E303K^ and Ednra^Y129F^.

A previous report suggested that the Y129F mutation of *EDNRA* can significantly increase affinity for ET3 (32, 34), which is expressed in the maxillary and mandibular processes of E10–12 mouse embryos (32, 35). To confirm both ET1 and ET3 expression in the developing face at a stage critical for the determination of maxillomandibular identity, we performed quantitative reverse–transcription PCR (q-RT-PCR) on embryos at E9.5. In contrast to the preferential expression of ET1 in the mandibular process, ET3 expression was detected at similar levels in both maxillary and mandibular processes (Figure 2K).

The above results suggest that the MFDA phenotype is due, at least for *Ednra^Y129F^*, to ectopic activation of mutant ET_A_R by ET3 in the maxillary region. To test this possibility, we introduced an *Edn3*-null allele into both mutant mice using the CRISPR/Cas9 system. Remarkably, the heterozygous *Edn3*-null allele mostly rescued both mutant phenotypes, except for the deformity of the incus and the eyelid dysplasia (Figure 2, E–H, Supplemental Figure 3, A and B, and Table 1). When both *Edn3* alleles were deleted, the morphology of the incus was completely normalized in each heterozygous *Ednra* mutant (Figure 2I and Table 1), while open eyelids at birth was incompletely rescued by *Edn3*^–/–^ in the ET_A_R-E303K mutants (Table 1). *Edn3*^–/–^ also rescued most phenotypes associated with each homozygous *Ednra* mutant (Supplemental Figure 3, C and D, and Table 1). Our results indicate that the gain-of-function phenotypes associated with the ET_A_R mutants are almost entirely dependent on ET3 (Figure 2L), but that the E303K mutation may increase basal or ET3-independent ET_A_R activity, particularly in the context of eyelid development (Supplemental Figure 1B and Table 1).

### ET_A_R-Y129F and -E303K enhance ET3-induced ERK phosphorylation and intracellular Ca2^+^ mobilization in HeLa cells.

**Figure 3:**
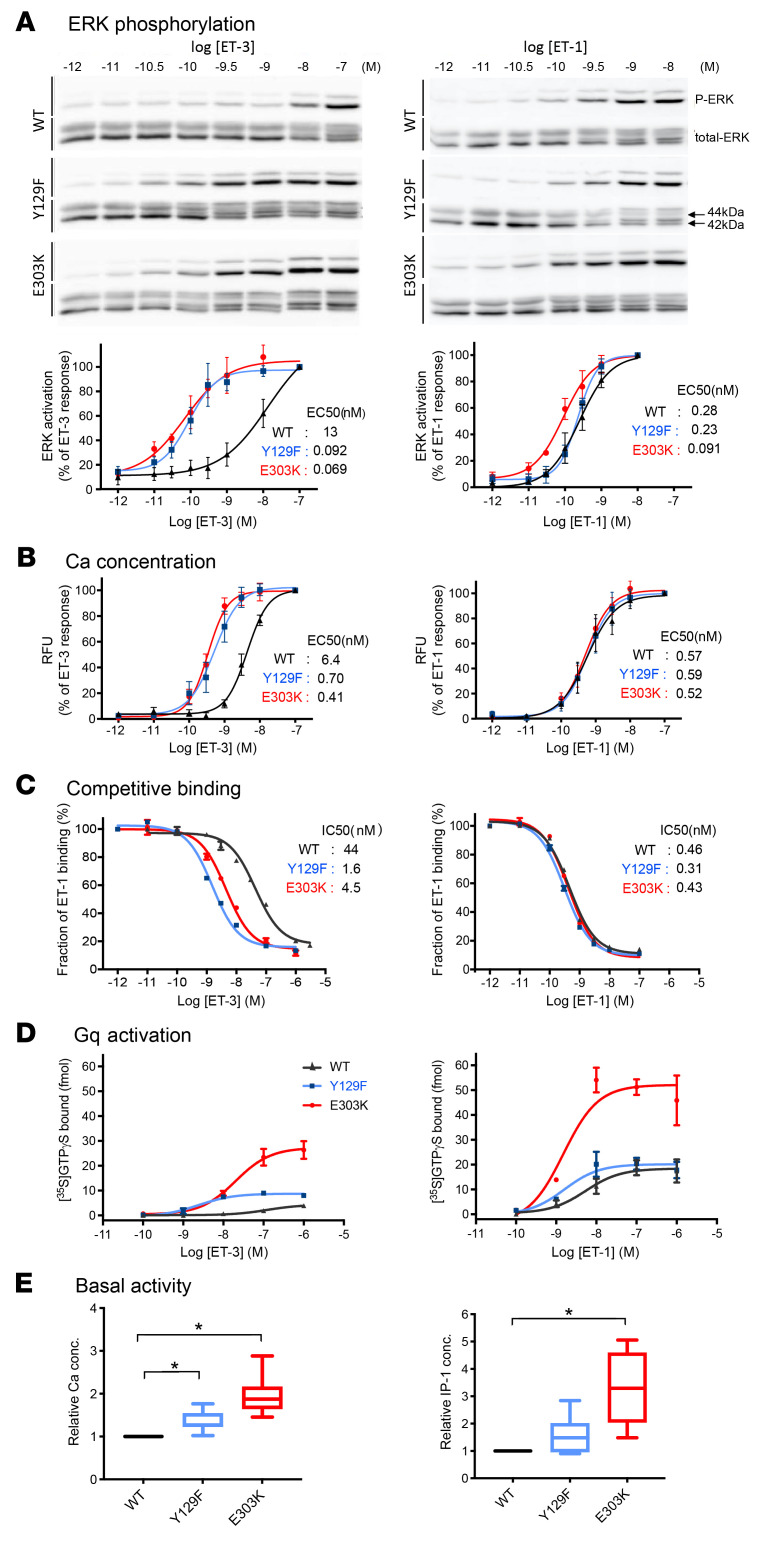
Gain-of-function properties of the ET_A_R mutants are caused by their ligand-binding affinity and Gq activation. (**A**) ERK phosphorylation assay. Representative Western blots (upper) and dose-response profiles (lower) of phosphorylated ERK (p-ERK) relative to total ERK, stimulated with ET3 (left) or ET1 (right). *n* = 3 (**B**) Intracellular calcium mobilization assay. Dose-dependent elevation of intracellular calcium concentrations stimulated by ET3 (left) or ET1 (right). *n* = 4. RFU; relative fluorescent unit. (**C**) Competitive ligand–binding assay. Competition-equilibrium binding was performed on crude membrane extracts using [^125^I] ET1 and the cold competitor agonist ET3 (left) or ET1 (right). Each value represents the mean ± SEM (also see Supplemental Table 4) from 2–3 independent experiments performed in duplicate. (**D**) Gq activation assay. Gq protein and Flag-tagged ET_A_R were reconstituted in rHDL particles and [^35^S] GTPγS binding was measured in the presence of ET3 (left) or ET1(right). Each value represents the mean ± SEM from 2–3 independent experiments performed in duplicate. (**E**) Measurement of basal activity. Intracellular calcium concentrations (left, *n* = 11) and IP_1_ concentrations (right, *n* = 6) in WT or mutant ET_A_R–expressing HeLa cells. Steel-Dwass’s multiple comparison test, *P < 0.05.

We showed previously that G_q/11_ is responsible for the specification of mandibular identity by ET1-ET_A_R signaling (36). Using HeLa cells overexpressing WT or mutant ET_A_Rs, we next examined the effects of ET ligands on phosphorylation of ERK, which is a key effector for signaling downstream of G _q/11_. Western blotting revealed that both ET_A_R-Y129F and E303K increased the sensitivity to ERK phosphorylation to ET3 by more than 2 orders of magnitude (Figure 3A left). Remarkably, the E303K mutation also enhanced ET1-induced ERK phosphorylation by approximately 3-fold, whereas the ET_A_R-Y129F did not affect the sensitivity to ET1 (Figure 3A, right). ERK phosphorylation can also be attributed to activation of other G proteins and recruitment of arrestin during GPCR signaling (37–39). We therefore evaluated Ca^2+^ mobilization and phospholipase C-mediated hydrolysis of inositol phospholipids, which represent more specific downstream readouts of G_q/11_ signaling. To estimate inositol hydrolysis, we measured concentrations of inositol 1 phosphate (IP_1_), which is a stable metabolite of inositol 1, 4, 5-triphosphate (IP_3_). ET_A_R-Y129F and E303K showed enhanced intracellular Ca^2+^ and IP_1_ concentrations in response to ET3, as estimated by the EC_50_ (Figure 3B left and Supplemental Figure 4A), whereas no difference was found in the dose response to ET1 (Figure 3B right and Supplemental Figure 4B). The response of ET_A_R -E303K to ET1 enhancement in the phosphorylated ERK assay compared with ET_A_R-WT and ET_A_R-Y129F, and no difference in response to the observation of ET1 in the Ca and IP_1_ assays, may suggest enhanced GPCR kinase (GRK) phosphorylation and arrestin recruitment for ET_A_R-E303K. Regarding the effects of other substitutions at E303, arginine (R) enhanced the response to ET3 as much as K, whereas aspartic acid (D) did not (Supplemental Figure 5, A and B). Replacement of E303 by neutral amino acids alanine (A) or glutamine (Q) resulted in partial enhancement (Supplemental Figure 5, A and B). These results indicate that mutations at Y129 and E303 can enhance G_q/11_-mediated signaling in response to ET3, and that the charge of the residue at position 6.32 may be critical.

### ET_A_R-Y129F and E303K confer high affinity ET3 binding on ET_A_R.

The above findings in mouse models and in vitro experiments indicate that, in addition to Y129, which is near the ligand-binding pocket, E303 may also serve as a determinant of ligand binding affinity even though it is located on the intracellular side of TM6. To explore this possibility, we performed ligand binding assays using crude membrane extracts from HEK293 cells with WT or mutant expression vectors. Competitive binding assays showed similar IC_50_ values for ET1 binding in WT and mutant receptors (Figure 3C). By contrast, the binding affinity to ET3 was increased in ET_A_R-Y129F and ET_A_R-E303K by approximately 27 and 10 fold, respectively (Figure 3C). To minimize the effect of associated proteins such as trimeric G proteins, we performed competitive ligand binding assays using WT and mutant receptor preparations incorporated into a reconstituted high-density lipoprotein (rHDL) phospholipid bilayer particle (Nanodisc). The ET_A_R-Y129F and ET_A_R-E303K mutants increased ET3 binding affinity by approximately 27 and 5 fold, respectively, in qualitative accordance with experiments using crude membrane extracts (Supplemental Figure 6).

### Differential effects of ET_A_R-Y129F and E303K on G protein activation and basal activity.

That the above findings showed similar activation of downstream signaling pathways in both mutants, although ET_A_R-E303K increased ET3 binding affinity less effectively than ET_A_R-Y129F, led us to speculate that ET_A_R-E303K might potentiate G protein activation upon ligand binding. To test this possibility, we incorporated WT and mutant receptor proteins into rHDL particles together with Gα_q_ and βγ subunits. After normalization of rHDL-ET_A_R quantities by measuring binding to equivalent amounts of radioactively labeled ET1, we evaluated G protein activation by [^35^S]GTPγS binding. ET_A_R-E303K largely increased Gα_q_ activation efficacy upon stimulation by ET-1 as well as ET-3, whereas, to a lesser extent, ET_A_R-Y129F increased only ET3-induced Gαq activation (Figure 3D). Thus, the increased ET3-dependent Gαq activation of ETAR-Y129F mainly reflects enhanced ET3 binding affinity, whereas that of ET_A_R -E303K reflects both enhanced ET3 affinity and a high intrinsic tendency toward activation.

To further test the gain-of-function properties of ET_A_R-Y129F and ET_A_R-E303K, we evaluated basal activity by measuring intracellular Ca^2+^ and IP_1_ levels without ligand, as an index of G_q_ activity. ET_A_R-E303K and, to a lesser extent, ET_A_R-Y129F, exhibited an increase in intracellular IP_1_ and/or Ca^2+^ concentrations compared with ET_A_R -WT without any difference in maximum response (Figure 3E and Supplemental Figure 7), indicating increased basal G_q_ activity. Taken together, these results suggest that both mutants have gain-of-function properties with different dependence on ligand-binding affinity.

### MD simulations reveal dynamic conformational changes in ET_A_R-E303K.

Structural analysis could shed light on the mechanisms underlying the gain-of-function properties of mutant ET_A_R, but no experimental structures are currently available for the WT receptor. As the conformation of membrane proteins fluctuates constantly regardless of ligand binding, we established a mouse ET_A_R homology model under membrane–water layer conditions, using the crystallographic structure of human ET_B_R (PDB 5GLI) as a template, in order to perform MD simulations (Supplemental Methods). The amino acid identity between mouse ET_A_R and human ET_B_R is 62 % as a whole and 71.5% from helices 1–8, which is considered sufficient for prediction of target structures, given that reliable homology modeling requires sequence identity higher than 35%–40% between target and template (40, 41).

**Figure 4:**
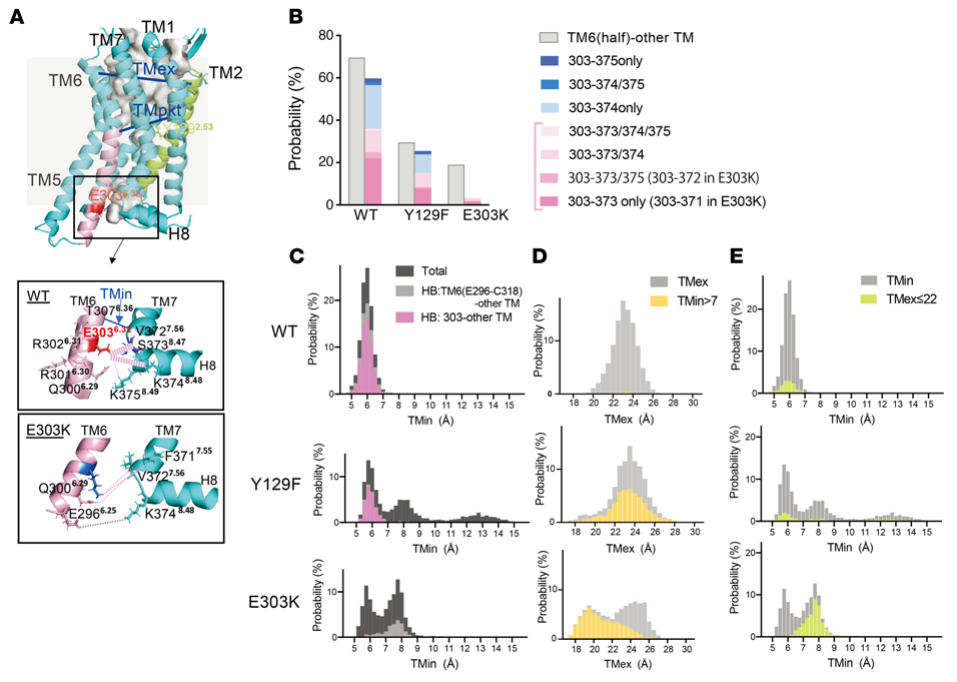
Loss of hydrogen bonds increases flexibility of TM6/7 in ET_A_R-E303K. (**A**) Homology modeling of the ligand-free inactive form of mouse ET_A_R (apo ET_A_R). 3D visualizations were generated using PyMOL. The cytoplasmic TM6/7 and H8 regions of ET_A_R-WT and ET_A_R-E303K are magnified in boxes below. The cytoplasmic half of TM6 (E296^6.25^ to C318^6.47^) is shown in pink. TM2 is denoted in lime. TMin, TMpkt and TMex represent distances between T307^6.36^Cα and V372^7.56^Cα, between D133^2.57^Cα and W319^6.48^ Cα, and between L141^2.65^Cα and K329^6.58^ Cα, respectively. Broken pink lines indicate HBs between the side chain of the 303 residue (E or K) and other TM residues, with width reflecting each HB probability. (**B**) Hydrogen bonding between cytoplasmic half of TM6 or E303 and other regions. Probability of HB formation between the cytoplasmic half of TM6 (from E296 to C318) and other TMs (left columns, grey), or between the 303 residue (E or K) and other TMs (right columns, colored differently according to HB partners), for ET_A_R-WT, -Y129F and -E303K. The probability is defined as the number of snapshots having HBs in a total of 3,000 shots (%). (**C**) Probability distribution of TMin. HBs formed between the cytoplasmic half of TM6 (from E296 to C318) and other TMs (light gray) and between the 303 residue and other TM residues (pink) are superimposed on total TMin (dark gray). (**D**) Probability distribution of TMex. Total TMex (light gray), and TMin > 7 Å (yellow) are superimposed. (**E**) Probability distribution of TMex < 22 Å (lime) superimposed on total TMin distribution (light gray).

Analysis of apo ET_A_R-WT (i.e., ET_A_R-WT without ligand) MD simulation revealed that most of the hydrogen bonds (HBs) between the intracellular half of TM6 and other TMs were formed through E303^6.32^. In particular, it forms HBs with S373^8.47^ and/or K374^8.48^ located at the junction of TM7 and helix 8 (H8) with high probability (Figure 4, A and B, and Supplemental Figure 8A). On the other hand, the positively charged mutant K303^6.32^ had little probability of forming HBs with other TMs. When the TM6–TM7 distance on the intracellular side was evaluated as the distance between T307^6.36^Cα atom and V372^7.56^Cα atom (hereafter referred to as TMin), it showed a unimodal distribution (5–7 Å) for ET_A_R-WT, and a multimodal distribution (5–7 Å and > 7 Å) for each mutant (Figure 4, A and C). When HBs were formed between E303 and other TMs, they were restricted within the short TMin peak (5–7 Å) for ET_A_R-WT and ET_A_R-Y129F (Figure 4C pink). Thus, the intracellular end of TM6 harboring E303K becomes more flexible because of the reduction of HBs due to the substitution of lysine by glutamic acid. Accordingly, upon deletion of the HB between E303 and S373 by replacing the serine of the latter with glutamic acid, the helix also became more flexible, with a bimodal distribution of TMin as seen for E303K (Supplemental Figure 8, B and C), indicating the importance of E303-associated HBs for regulating the flexibility of TM6.

**Figure 5:**
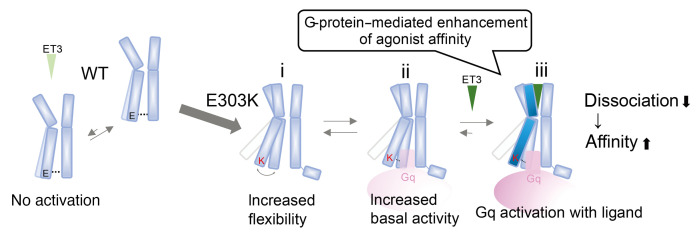
Presumed mechanism for enhanced affinity of ET_A_R-E303K to ET3. (i) Increased flexibility of TM6 due to E-to-K substitution at residue 303. (ii) Increased basal activity. (iii) G protein–mediated enhancement of agonist affinity; upon entering the ligand binding pocket, ET3 is prevented from egressing due to the narrowing of the ligand binding pocket, leading to increased ET3 affinity and sustained Gq activation.

The distance between TM2 and TM6 on the extracellular side, represented by the distance from L141^2.65^Cα to K329^6.58^Cα (hereafter referred to as TMex), showed a unimodal distribution for ET_A_R-WT and ET_A_R-Y129F, but was bimodal for ET_A_R-E303K (Figure 4, A and D). When TMin was greater than 7 Å for ET_A_R-E303K, it was distributed mainly in the shorter peak of TMex (Figure 4D yellow), while TMex less than 22 Å was distributed mainly in the larger peak of TMin (Figure 4E lime), indicating that the conformation of the intracellular side influenced that of the extracellular side in ET_A_R-E303K. Importantly, the wide TMin state is predicted to be capable of coupling to G protein without ligand binding during the dynamic and flexible motion of mutant ET_A_R, resulting in increased basal activity (3) (Figure 5). To assess the broader relevance of this, we estimated the distance corresponding to TMin (amino acid positions 6.36–7.56) in several class A GPCR crystal structures from the Protein Data Bank and observed that the active and inactive states of GPCRs appear to correspond to long (7.7–11.5 Å) and short (5.5–6.7 Å) distances, respectively (Supplemental Table 2). These data suggest that ET_A_R-E303K preferentially adopts the active state regardless of ligand availability, allowing G protein binding and increased basal activity, which itself allosterically affects the conformation of the ligand binding domain (Supplemental Figure 9).

### Reduced water network contributes to the acquired ET3 affinity in ET_A_R-Y129F.

**Figure 6:**
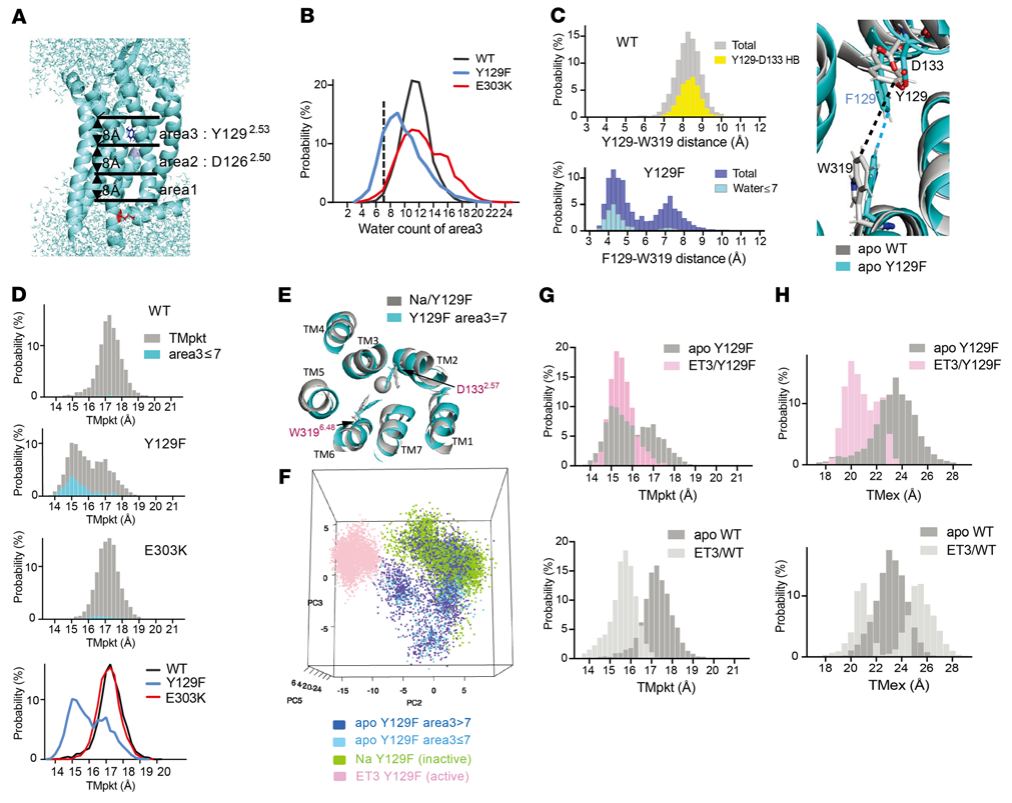
Apo ET_A_R-Y129F takes on a form intermediate between inactive and active states due to a reduced water network. (**A**) Division of the interhelical cavity beneath the ligand binding site into 3 regions of 8 Å each. Area 1 is the G protein binding area; area 2 contains D126^2.50^; and area 3 contains Y129^2.53^. (**B**) Probability distribution of the numbers of water molecules in area 3. The dashed vertical line indicates the point at which the water number is 7. (**C**) Distance between apo Y/F129^2.53^CZ atom and W319^6.48^ CH2 atom. The representative side chains are superimposed (right). (**D**) Probability distribution of TMpkt for apo ET_A_R-WT, -Y129F and -E303K. The distributions when area 3 contains ≤ 7 water molecules are overlaid (sky blue). The lowest panel is a stack of TMpkt distributions for ET_A_R-WT (black), -Y129F (blue), and -E303K (red). (**E**) TMpkt level structure. A representative apo ET_A_R-Y129F with area 3 = 7 water molecules (sky blue) is superimposed on Na-bound ET_A_R-Y129F (inactive state; grey). (**F**) PCA for the conformation of the extracellular half of TMs 2, 6, and 7 of ET_A_R-Y129F in different states. A snapshot of the 3D representation of the PC2-PC3-PC5 axis. This is available in a 3D viewer file at https://kurihara-utokyo.github.io/yk2021-et/ (**G**) Probability distribution of TMpkt for ETAR-Y129F (upper) and ETAR- WT (lower), in the apo forms (dark grey) or bound to ET3 (pink or light grey). (**H**) Probability distribution of TMex for ETAR-Y129F (upper) and ETAR- WT (lower), in the apo forms (dark grey) or bound to ET3 (pink or light grey).

Next, we examined how ET_A_R-Y129F increases ligand binding affinity. Y129^2.53^ in ET_A_R is located in the putative Na^+^/water pocket under the orthosteric ligand binding pocket. As Y129^2.53^ is expected to form HBs with water molecules through its hydroxyl group (Supplemental Figure 10A), substitution with uncharged phenylalanine may disrupt the HB network in the Na^+^/water pocket and the conformation of adjacent helices. To evaluate the state of water molecules in the interhelical space under the ligand binding site, it was divided into 3 sections of 8 Å each; the G protein binding area (area 1), the D126^2.50^-area (area 2), and the Y129^2.53^-area (area 3) (Figure 6A). MD simulation showed a decreased number of water molecules in area 3 of ET_A_R-Y129F compared with that of ET_A_R-WT and -E303K (Figure 6B). Y129^2.53^ forms a HB with D133^2.57^ 1 turn above Y129 at about 50% frequency in the simulation thereby restricting the movement of the tyrosine side chain (Figure 6C). However, the phenylalanine side chain does not form HB and is more flexible because it moves widely (Supplemental Figure 11). It may compete with water molecules in the Na-water pocket lumen. The narrowed distance between Y/F129^2.53^ and W319^6.48^ in ET_A_R-Y129F indicates that the pocket tends to shrink (Figure 6C). These results suggest that the bulky hydrophobic side chain of F129 also reduces the number of water molecules.

When the distance of the main chain between D133^2.57^Cα and W319^6.48^Cα (hereafter referred to as TM pocket; TMpkt, Figure 4A) was regarded as an index of the conformation of the Na^+^/water pocket, it showed unimodal distribution for ET_A_R-WT or ET_A_R-E303K, and a bimodal distribution for ET_A_R-Y129F (Figure 6D). The narrow TMpkt peak for ET_A_R-Y129F largely corresponded to a small number of water molecules (≤ 7) in area 3 (Figure 6D), demonstrating a shrinkage of the TMpkt compared with the Na^+^-bound inactive state (Figure 6E). By contrast, TMin or TMex of apo ET_A_R-Y129F showed no apparent correlation with water molecule numbers (Supplemental Figure 10B).

We then performed further MD simulations with Na^+^-bound ET_A_R-Y129F (an inactive state) and ET3-bound ET_A_R-Y129F (an active state), compared with Na^+^-free apo ET_A_R-Y129F, and analyzed the movement of the extracellular portions of TM2, TM6, and TM7 which are reported to move inward upon ligand binding in ET_B_R (42–44), by principal component analysis (PCA) (Figure 6F). The validity of this PCA was confirmed by examining apo WT and mutant apo ET_A_Rs in a Na^+^-free state (Supplemental Figure 12, A–C). Apo ET_A_R-Y129F conformations with decreased number of water molecules (≤7) in area 3 (Figure 6F) are considered to be an intermediate state because they are located separately from the active or inactive forms. The ET3-bound forms of ET_A_R-Y129F and ET_A_R-WT have a narrower TMpkt (Figure 6G), which is similar to that of apo ET_A_R-Y129F with decreased water molecules (≤7) in area 3 (Figure 6D). The TMex is also narrower in ET3/ET_A_R-Y129F (Figure 6H) and in ET3/ ET_A_R-WT (Figure 6H), indicating that a narrow TMex is a feature of the active state, in addition to a narrow TMpkt.

**Figure 7:**
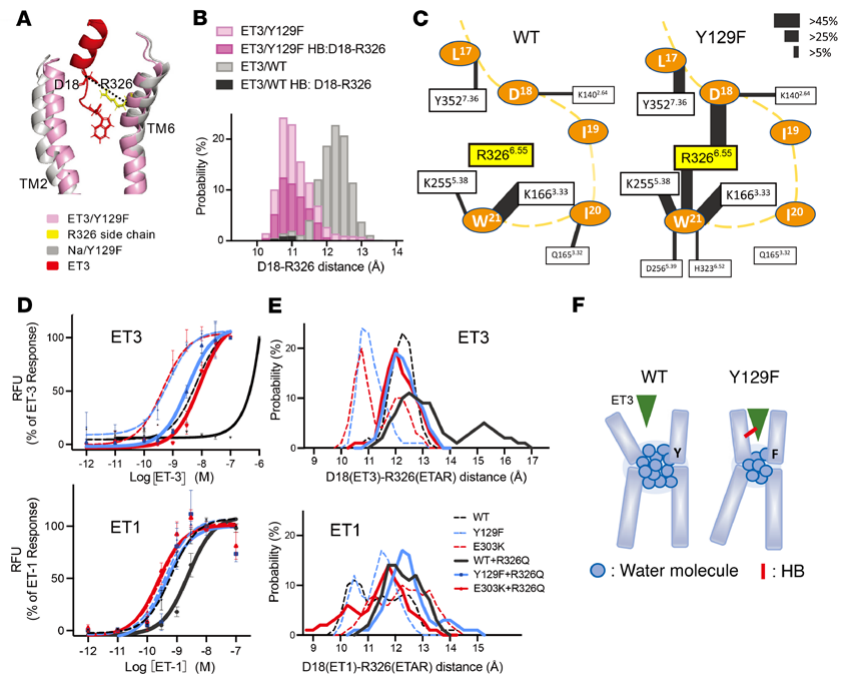
ET_A_R-Y129F prevents dissociation of ET3 by increasing probability of reduced distance between helices. (**A**) Comparison of representative structures of ET3 bound to ET_A_R-Y129F (active, pink) and Na-bound ET_A_R-Y129F (inactive, grey). Dashed line connects D18Cα of ET3 and R326Cα of ET_A_R-Y129F. (**B**) Probability distribution of distances between D18 of ET3 and R326 of ET_A_R-WT or -Y129F (grey and light pink, respectively), overlaid with the distribution of probabilities of hydrogen bonding between these residues in each case (dark pink for ET_A_R-Y129F and black for ET_A_R-WT). (**C**) Schematic representation of HBs between ET3 residues (orange ovals) and TM residues of ET_A_R (rectangles). The solid lines between pairs of residues indicate the presence of HBs, and the thickness of the line represents the probability of the HB. HB probabilities of less than 5.00% are omitted, including 4.00% for R326-W21 and 2.27% for R326-D18 for ET_A_R-WT. Yellow dashed line shows the main chain of the ET3 peptide. (**D**) Intracellular calcium mobilization assay in HeLa cells. R326Q mutants are shown in bold lines. Changes in calcium concentrations in response to each ET3 (upper) or ET1 (lower) concentration are plotted as mean ± SEM (also see Supplemental Table 4) from more than 4 independent experiments. RFU, relative fluorescent unit. (**E**) The distance between D18 Cα of ET3 (upper) or ET1 (lower) and R/Q326 Cα of ET_A_Rs. R326Q mutants are shown in bold lines. (**F**) Presumed mechanism for the enhanced affinity of ET_A_R-Y129F for ET3. The number of water molecules within the water network around Y129 decreases upon Y-to-F substitution, thereby increasing the probability of a state with a narrowed ligand pocket, leading to increased hydrogen bonding with ET3.

In order to further examine the effect of a narrow TMex, we measured the distance between ET3 and ET_A_R, represented by the distance from D18 Cα of ET3 to R326 Cα of ET_A_R (Figure 7A). It was shorter in ET_A_R-Y129F than in ET_A_R-WT (Figure 7B), and the probability of HB formation between ET3 and ET_A_R-Y129F was high, in particular for the D18-R326 and W21-R326 interactions (Figure 7C and Supplemental Figure 13, A and B). This would have a significant effect on ET3 binding affinity, in other words, substituting tyrosine with phenylalanine narrows the TMpkt due to the decreased number of water molecules in area 3, which leads to narrowing of TMex and formation of HBs. This could prevent the dissociation of ET3 from ET_A_R, thereby increasing ET3 affinity (Figure 7F). To verify the importance of HBs between R326^6.55^ in ET_A_R and residues in ET3 for increased ET3 affinity in ET_A_R mutants, we substituted R326 with glutamine in ET_A_Rs in order to eliminate those HBs. As expected, R326Q substitution in ET_A_R -Y129F and ET_A_R-E303K led to a loss of HBs between Q326 and either D18 or W21 of ET3 (Supplemental Figure 14A), resulting in ET3 affinity that was decreased to the level observed in ET_A_R-WT. Moreover, R326Q substitution in ET_A_R-WT almost abrogated responsiveness to ET3 (Figure 7D, upper). On the other hand, R326Q substitution did not affect intracellular Ca^2+^ mobilization in response to ET1 in ET_A_R -Y129F and ET_A_R -E303K, despite the loss of R326 HBs (Figure 7D, lower and Supplemental Figure 14B). These results indicate that HBs involving R326 are less critical in binding to ET1 than to ET3. The narrow peak of the R326Cα-D18Cα distance corresponds to ET_A_R activation by ET3 (Figure 7E, upper). However, in the case of ET1, this distance does not correlate with the activity; that is, HBs between R326 and ET1 are not essential for activation by ET1 (Figure 7E, lower).

## Discussion

### MFDA is due to ET_A_R gain-of-function mutations.

Following the identification of 2 different ET_A_R missense variants, Y129F and E303K, in MFDA, we report here another patient with MFDA harboring the E303K mutation in a mosaic state and mouse models carrying the ET_A_R-Y129F or -E303K mutations, which mostly recapitulate morphological manifestations of MFDA. Strikingly, the majority of features observed in the mutant mice were rescued by heterozygous loss of *Edn3*, while the incus required homozygosity for the *Edn3*-null allele to recover its normal morphology. The variable gene dosage effect may reflect a regional difference in the intensity of ET3 expression or sensitivity to activation for normal development. This result, taken together with our previous finding on G protein selectivity (36), suggests that the ET_A_R-Y129F and -E303K phenotypes are caused by ET3-dependent gain-of-function mechanisms through the activation of Gα_q/11_. Indeed, both mutations enhanced signaling pathways downstream of Gα_q_ in response to ET3, as assayed by intracellular calcium concentration, to a similar extent. By contrast, ligand binding assays and G protein activation assays indicated different mechanisms underlying the enhanced signaling for each mutation.

Some differences between patients and mouse models are noted. The most significant difference is alopecia in patients with MFDA but not in mouse models. Also, the mandible (especially the proximal region) is more dysplastic in patients with MFDA than the mutant mice (32). These differences may indicate that the extent and/or effect of ET3-induced ET_A_R hyperactivation is different between species. In addition, it is surprising that some manifestations of the patient harboring a p.E303K mutation in the present report are no less severe than the mouse phenotype in spite of low-level (8–11%) mosaicism, as estimated from peripheral blood DNA. Similarly, no clear differences in phenotypic severity were noted between patients with MFDA harboring a mosaic versus heterozygous p.Tyr129Phe mutation (32). Although tissue mosaicism rates can be variable in different tissues (45), it suggests that ET_A_R activation in only a small number of cells may be sufficient for the manifestation of gain-of-function phenotypes.

### Mechanism of gain-of-function of the ET_A_R-E303K mutation.

Our MD analysis revealed different mechanisms underlying gain-of-function in the E303K and Y129F ET_A_R mutants. ET_A_R-E303K is characterized by the combination of increased ET3 binding affinity and facilitated Gα_q_ activity, to which changes in the intrahelical HBs are likely to contribute. In some class A GPCRs, an acidic residue at position 6.30 interacts with R^3.50^ within the conserved aspartate-arginine-tyrosine (DRY) motif, creating a HB-mediated ionic lock to stabilize the inactive state, and this ionic lock is broken during receptor activation (6, 7, 46–48). ET_A_R cannot make this TM3-TM6 ionic lock because the residue at position 6.30 is basic arginine (R301) (Supplemental Table 3), so R183^3.50^ forms a HB primarily with D182^3.49^(39%; data not shown). Instead, ET_A_R-WT forms the nonclassical ionic lock between TM6 and the TM7-H8 linker (S373^8.57^ or K374^8.58^, Figure 4, A and B), and the disruption of this ionic lock in ET_A_R-E303K produces a bimodal distribution of TMin (Figure 4C) which most likely reflects transitions between populations of inactive (intact ionic lock) and active states (broken ionic lock) of the receptor. This propensity to reside in the active state would explain the enhanced sensitivity in all the signaling assays (Figure 5).

In addition, lysine substitution at position 6.32 increases the positive charge of the Gα-binding pocket (Supplemental Figure 15A). Basic residues at this position are involved in the interaction with Gα through HBs, for example, bonds between R^6.32^ of the μ-opioid receptor and H5-L25 of the α5-helix in Gα_i_, and between K^6.32^ of β_2_AR and H5-E24 of Gα_s_ (4, 50, 51). Considering the structural similarity of the H5 domain between Gα_q_ and other Gα proteins (Supplemental Figure 15B), K303^6.32^ may strengthen the interaction with Gα_q_, leading to increased basal activity. Regarding the increased affinity to ET3, our MD simulation provides insights by showing a reciprocal linkage between the intracellular and extracellular sides of TM6 in ET_A_R-E303K, which is considered an allosteric effect on ligand binding. In the ternary-complex model of GPCR dynamics (Supplemental Figure 9), GPCRs adopt closed-active, high-affinity structures that hinder dissociation of the bound ligand in the presence of an agonist. G protein or active nanobody coupling to GPCRs also influences ligand binding affinity by inducing allosteric conformational changes of the orthosteric ligand binding site, leading to a closed-active conformation with narrowing of the extracellular side of TMs, even without ligands (3, 6, 7, 51). The E303^6.32^K substitution may increase the affinity of the orthosteric ligand binding site through a similar allosteric effect, which is potentiated by the increased basal G protein binding and activation. Thus, ET3/ET_A_R-E303K signaling may involve the mechanism of G protein–mediated enhancement of agonist affinity.

### Mechanism of gain-of-function of the ET_A_R-Y129F mutation.

Several class A GPCRs have a Na^+^/water pocket composed of ionic binding between D^2.50^ and a Na^+^ and surrounding water molecules (8, 9, 11). Y129^2.53^ is located 1 turn above D126^2.50^ and participates in the Na^+^/water network that maintains an inactive state. In our MD simulation with Na^+^, the sodium ion is retained by ionic bonds with D126, S362, and/or T172 (Supplemental Figure 16). The triple ionic bonds are decreased in ET_A_R-Y129F, but at least 1 or 2 ionic bonds are almost always present in this mutant, so phenylalanine substitution may not affect sodium retention. On the other hand, removing the hydroxy group through phenylalanine substitution decreases the number of water molecules constituting the Na^+^/water pocket, which increases the propensity to collapse the Na^+^/water pocket. This water decrease is accompanied by a lateral-inward shift of the extracellular portions of the TMs, increasing ET3 binding affinity by inhibiting dissociation from the binding pocket (Figure 7F). However, we could not rule out the possibility of the outward shift of the intracellular half of the TMs because we used the intermediate rather than the active state of ET3-bound ET_B_R (PDB:6IGK) as an available ligand-bound template. This template is restricted in the movement of the intracellular side of the TMs because of the large T4-lysozyme protein within intracellular loop 3 and several mutations for thermal stabilization. Although MD simulations of ET_A_Rs were performed excluding the T4-lysozyme protein, it remains possible that the movement of the intracellular side of active ET_A_R bound to ET3 was not adequately simulated.

Another caveat for the inference that a HB between Y129^2.53^ and D133^2.57^ could contribute to the water retention by removing bulky side chain from the water pocket space is the use of the ET_B_R template in which D154^2.57^ (corresponding to D133^2.57^ of ET_A_R) is substituted by alanine, which may be a limitation when the side chain of D133^2.57^ is simulated. Despite these limitations, MD simulations and experimental verification support the conclusion that the inward shifts of the extracellular portion of the TMs were considered to favor ET3 binding through interacting forces such as van der Waals forces and Coulomb’s force, given the relatively short distance between D18 of ET3 and R326^6.55^ of ET_A_R-Y129F.

ET_A_R-Y129F and ET_A_R-E303K showed increased affinity and responsiveness to ET3 due to allosteric influences from outside the ligand pocket, whereas ET1 showed no further pharmacological affinity enhancement. ET1 binding is possibly affected by the lid consisting of the N-terminal and extracellular loop as shown in ET_B_R(42–44), since the affinity for ET1 was well maintained upon the loss of HBs with R326, which, in contrast, is critical for ET3-ET_A_R binding. Thus, ET1 and ET3 clearly differ in the mechanism of affinity for the same receptor.

Our findings provide insight into how allosteric communication within GPCRs can affect ligand binding affinity, and how alteration of normal GPCR function can cause human diseases. Furthermore, the molecular details of ET_A_R structure and function uncovered here provide a basis for rational drug design aimed at manipulating endothelin receptor activity relevant to more frequent diseases, such as pulmonary hypertension.

## Methods

### Patient genetic studies

The patient’s clinical details are listed in Supplemental Table 1. Genomic DNA from the patient was screened for copy number variations using a Nimblegen 135K comparative genomic hybridization microarray with a resolution of 100 kb. For next-generation sequencing, genomic DNA was extracted from the patient’s blood, captured using a custom-designed Agilent SureSelect kit, and sequenced on an Illumina NextSeq 500 machine. Analysis of a subpanel of genes implicated in craniofacial malformations indicated the presence of the *EDNRA* variant c.907G>A at a frequency of 8%, from a total read count of 280 at this site. To further confirm the percentage of mosaicism, sequencing was repeated in 2 independent experiments using either a Twist library protocol (Twist library preparation EF kit and Twist CD index adapters) or an Agilent library protocol (SureSelect XT HS2 Library preparation and Enzymatic Fragmentation kits and index primer pairs), with a SureSelect XT HS2 Target Enrichment Kit used for capture in both experiments. Independently of the calling program used — and with or without the use of unique molecular indexing to avoid duplicated reads — these tests repeatedly gave an *EDNRA* variant read frequency of 10%–11%. Sanger sequencing of the *EDNRA* variant was performed according to standard techniques. For the colony PCR experiment, the *EDNRA* PCR product was amplified from the patient’s peripheral blood DNA and was cloned into pCRII-TOPO (Invitrogen), which was then used for bacterial transformation, followed by PCR and Sanger sequencing of individual colonies.

### Mutant mice

Mice were housed with 2–5 individuals per cage in SPF conditions in an environmentally controlled room at 23˚C ± 2˚C. Relative humidity was kept at 50%–60% and cages were kept under a 12 hour light and 12 hour dark cycle.

Mice carrying a Y129F or E303K substitution in the *Ednra* gene and *Edn3* knockout mice were generated using the CRISPR/Cas9 system by zygote injection (52, 53). A mixture of Cas9 mRNA (transcribed by mMESSAGE mMACHINE T7 ULTRA kit with p3s-Cas9HC plasmid as a template), guide RNA (transcribed by the MEGAshortscript T7 kit using the DR274 plasmid containing the target sequence as template), and mutant oligonucleotides were injected into the pronuclei of fertilized ICR mouse eggs to generate a 1-base substitution in Y129F and E303K mutants. To generate *Edn3* knockout mice, 2 PAM sequences were chosen around the mature ET3-coding region, in order to achieve complete deletion of mature ET3. Sequences of guide RNA target sites and oligonucleotides are available in Supplemental Table 5. The fertilized eggs injected with mRNAs with and without oligonucleotides were implanted into pseudo-pregnant mice. Mice were bred on the background of Crl:CD1(ICR) from Charles River Laboratories, Japan. Double mutant mice were obtained by crossing double-heterozygous (*Ednra^E303K/+^;Edn3^+/–^* and *Ednra^E303K/+^;Edn3^+/–^)* mice, and their phenotypes were compared among littermates with different genotypes.

### ETAR structural studies: system setup and MD simulation

An initial structure of the mouse ET_A_R was constructed by homology modeling using the ligand-free form of human ET_B_R (PDBID: 5GLI) as a template, because no crystal structures of mouse ET_A_R have been reported to date. Sequence identity between mouse ET_A_R and human ET_B_R is 62% (71.5% for the helix domains). A multiple-sequence alignment of mouse, rat, and human ET_B_R and ET_A_R was built using T-COFFEE (54). As the template structure, the rerefined and rebuilt structure of human ET_B_R deposited in the PDB-REDO databank (ID: 5GLI) was employed, and the homology modeling was executed by MODELLER (55). In the modeling process, the fused T4L was deleted. Since we focused on dynamics of typical GPCR structures consisting of 7 transmembrane helices (TM1–7) and intracellular helix 8, the corresponding residues 65–385 of the ET_A_R model structure were used in MD simulations. A complex system of the model ET_A_R structure embedded in membrane and water molecules was constructed by the membrane builder implemented in CHARMM-GUI (56). The protonation states of protein were determined by p*K*_a_ using the program H++. The orientation of ET_A_R relative to the lipid bilayer was set to that of the ET_B_R structure deposited in the orientations of proteins in membrane (OPM) database (57). The type of the lipid bilayer was 1-palmitoyl-2-oleoyl-sn-glycero-3-phosphocholine (POPC). The mutations E303K, Y129F, S373E, or R326Q were substituted using the setup process in CHARMM-GUI. In the Na-bound ET_A_R model, the Na^+^ ion in the Na/water pocket was modeled so that the interaction mode around the Na^+^ ion, including crystal water molecules formed in the crystal structure of the human A_2A_ adenosine receptor (PDBID: 4EIY), was reproduced. An ET3 or ET1-bound ET_A_R model was constructed by homology modeling using ET3 or ET1-bound human ET_B_R (PDBID: 6IGK for ET3; PDBID: 5GLH for ET1) as a template. The membrane-water systems for the mutant, Na-bound or ET-bound ET_A_R models were constructed using the same procedure described above.

MD simulations were performed using the MD program package GROMACS ver. 2016.3 (58) with the CHARMM36 force field for proteins (59, 60) and membranes (61, 62) and the TIP3P water model. After energy minimization and equilibration runs, production runs were performed. The isothermal-isobaric (NPT) ensemble was adopted. The temperature and pressure were set at 300 K and 1 atm. Three 1 μs production runs were performed independently for each system. The 3 runs started from the same initial system prepared after the equilibration runs. In the production runs, no restraints were imposed. Trajectory analyses were performed using snapshots saved every 1 ns in each run (3,000 snapshots in each system). Interatomic distance and HBs were detected using visual MD (VMD) (63).

### Other methods

Further information about the methodology is included in Supplemental Methods.

### Data availability

All input files for the MD simulations have been deposited in GitHub (https://github.com/IkeguchiLab/mETAR-MD, branch name: main, commit ID: d9c50554c2c48187467966b30f40bc6916702ce9).

### Statistics

Mann-Whitney U test was performed to determine the significance for ET1/3 concentration, and Steel-Dwass’s multiple comparison test was used for basal activity and maximal calcium concentration. Data were calculated by Prism8 and expressed as mean ± SEM, except where noted.

### Study approval

#### Animal study.

All of the animal experiments were performed in accordance with the guidelines of the University of Tokyo Animal Care and Use Committee with approval from the IRB (approval number M-P19-050).

#### Patient and sample collection.

Written informed consent for genetic testing and for publication of patient images was obtained from the patient’s family. Human genetic research in this study was performed with approval from the institutional review board “Comité de Protection des Personnes Ile-deFrance II” (Necker Hospital); approval received 10th June, 2015.

## Author contributions

YK and HK conceptualized the project. YK, TE, CTG, YU, RS, TK, AI, RK, RA, and VP performed experiments. JA provided resources. YK, TE, CTG, JA, and HK wrote the original draft of the manuscript. YK, TE, CTG, JA, MI, and HK reviewed and edited the manuscript. YK, MI, JA, and HK acquired funding for the project.

## Supplementary Material

Supplemental data

## Figures and Tables

**Table 1:**
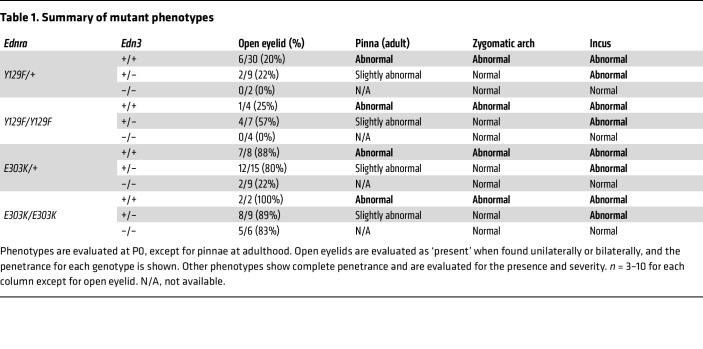
Summary of mutant phenotypes

## Version 1 01/13/2023

In-Press Preview

## Version 2 02/15/2023

Electronic publication

## References

[1] Farrens DL (1996). Requirement of rigid-body motion of transmembrane helices for light activation of rhodopsin. Science.

[2] Mahoney JP, Sunahara RK (2016). Mechanistic insights into GPCR-G protein interactions. Curr Opin Struct Biol.

[3] Manglik A (2015). Structural insights into the dynamic process of β2-adrenergic receptor signaling. Cell.

[4] Gregorio GG (2017). Single-molecule analysis of ligand efficacy in β_2_AR-G-protein activation. Nature.

[5] Rasmussen SG (2011). Crystal structure of the β2 adrenergic receptor-Gs protein complex. Nature.

[6] DeVree BT (2016). Allosteric coupling from G protein to the agonist-binding pocket in GPCRs. Nature.

[7] Staus DP (2016). Allosteric nanobodies reveal the dynamic range and diverse mechanisms of G-protein-coupled receptor activation. Nature.

[8] Katritch V (2014). Allosteric sodium in class A GPCR signaling. Trends Biochem Sci.

[9] Hori T (2018). Na^+^-mimicking ligands stabilize the inactive state of leukotriene B_4_ receptor BLT1. Nat Chem Biol.

[10] Ballesteros JA, Weinstein H (1995). Integrated methods for the construction of three-dimensional models and computational probing of structure-function relations in G protein-coupled receptors. Methods Neurosci.

[11] Venkatakrishnan AJ (2019). Diverse GPCRs exhibit conserved water networks for stabilization and activation. Proc Natl Acad Sci U S A.

[12] Yanagisawa M (1988). A novel potent vasoconstrictor peptide produced by vascular endothelial cells. Nature.

[13] Masaki T (2004). Historical review: endothelin. Trends Pharmacol Sci.

[14] Dhaun N, Webb DJ (2019). Endothelins in cardiovascular biology and therapeutics. Nat Rev Cardiol.

[15] Kurihara Y (1994). Elevated blood pressure and craniofacial abnormalities in mice deficient in endothelin-1. Nature.

[17] Kuratani S (2005). Craniofacial development and the evolution of the vertebrates: the old problems on a new background. Zoolog Sci.

[18] Minoux M, Rijli FM (2010). Molecular mechanisms of cranial neural crest cell migration and patterning in craniofacial development. Development.

[19] Santagati F, Rijli FM (2003). Cranial neural crest and the building of the vertebrate head. Nat Rev Neurosci.

[20] Kurihara Y (1995). Aortic arch malformations and ventricular septal defect in mice deficient in endothelin-1. J Clin Invest.

[21] Maemura K (1996). Sequence analysis, chromosomal location, and developmental expression of the mouse preproendothelin-1 gene. Genomics.

[22] Ozeki H (2004). Endothelin-1 regulates the dorsoventral branchial arch patterning in mice. Mech Dev.

[23] Ruest L-B (2004). Endothelin-A receptor-dependent and -independent signaling pathways in establishing mandibular identity. Development.

[24] Sato T (2008). An endothelin-1 switch specifies maxillomandibular identity. Proc Natl Acad Sci U S A.

[25] Tavares AL, Clouthier DE (2015). Cre recombinase-regulated endothelin1 transgenic mouse lines: novel tools for analysis of embryonic and adult disorders. Dev Biol.

[26] Rieder MJ (2012). A human homeotic transformation resulting from mutations in PLCB4 and GNAI3 causes auriculocondylar syndrome. Am J Hum Genet.

[27] Gordon CT (2013). Mutations in endothelin 1 cause recessive auriculocondylar syndrome and dominant isolated question-mark ears. Am J Hum Genet.

[28] Kanai SM (2022). Auriculocondylar syndrome 2 results from the dominant-negative action of PLCB4 variants. Dis Model Mech.

[29] Marivin A (2016). Dominant-negative Gα subunits are a mechanism of dysregulated heterotrimeric G protein signaling in human disease. Sci Signal.

[30] Clouthier DE (2013). Understanding the basis of auriculocondylar syndrome: insights from human, mouse and zebrafish genetic studies. Am J Med Genet C Semin Med Genet.

[31] Pritchard AB (2020). Loss-of-function of endothelin receptor type A results in Oro-Oto-Cardiac syndrome. Am J Med Genet A.

[32] Gordon CT (2015). Mutations in the endothelin receptor type A cause mandibulofacial dysostosis with alopecia. Am J Hum Genet.

[33] Tavares ALP (2017). Negative regulation of endothelin signaling by SIX1 is required for proper maxillary development. Development.

[34] Lee JA (1994). Tyr-129 is important to the peptide ligand affinity and selectivity of human endothelin type A receptor. Proc Natl Acad Sci U S A.

[35] Leibl MA (1999). Expression of endothelin 3 by mesenchymal cells of embryonic mouse caecum. Gut.

[36] Sato T (2008). Recombinase-mediated cassette exchange reveals the selective use of Gq/G11-dependent and -independent endothelin 1/endothelin type A receptor signaling in pharyngeal arch development. Development.

[37] DeWire SM (2007). Beta-arrestins and cell signaling. Annu Rev Physiol.

[38] Eichel K (2016). β-Arrestin drives MAP kinase signalling from clathrin-coated structures after GPCR dissociation. Nat Cell Biol.

[39] O’Hayre M (2017). Genetic evidence that β-arrestins are dispensable for the initiation of β_2_-adrenergic receptor signaling to ERK. Sci Signal.

[40] Kufareva I (2011). Status of GPCR modeling and docking as reflected by community-wide GPCR dock 2010 assessment. Structure.

[41] Schmidt T (2014). Modelling three-dimensional protein structures for applications in drug design. Drug Discov Today.

[42] Shihoya W (2016). Activation mechanism of endothelin ET_B_ receptor by endothelin-1. Nature.

[43] Shihoya W (2017). X-ray structures of endothelin ETB receptor bound to clinical antagonist bosentan and its analog. Nat Struct Mol Biol.

[44] Shihoya W et al (2018). Crystal structures of human ET_B_ receptor provide mechanistic insight into receptor activation and partial activation. Nat Commun.

[45] Qin L (2016). Detection and quantification of mosaic mutations in disease genes by next-generation sequencing. J Mol Diagn.

[46] Palczewski K (2000). Crystal structure of rhodopsin: a G protein-coupled receptor. Science.

[47] Ballesteros JA (2001). Activation of the β2-adrenergic receptor involves disruption of an ionic lock between the cytoplasmic ends of transmembrane segments 3 and 6. J Biol Chem.

[48] Schneider EH (2010). Impact of the DRY motif and the missing “ionic lock” on constitutive activity and G-protein coupling of the human histamine H4 receptor. J Pharmacol Exp Ther.

[49] Koehl A (2018). Structure of the μ-opioid receptor–G_i_ protein complex. Nature.

[50] Flock T (2017). Selectivity determinants of GPCR-G-protein binding. Nature.

[51] Warne T (2019). Molecular basis for high-affinity agonist binding in GPCRs. Science.

[52] Mashiko D (2013). Generation of mutant mice by pronuclear injection of circular plasmid expressing Cas9 and single guided RNA. Sci Rep.

[53] Wang H (2013). One-step generation of mice carrying mutations in multiple genes by CRISPR/Cas-mediated genome engineering. Cell.

[54] Di Tommaso P (2011). T-Coffee: a web server for the multiple sequence alignment of protein and RNA sequences using structural information and homology extension. Nucleic Acids Res.

[55] Jo S (2007). Automated builder and database of protein/membrane complexes for molecular dynamics simulations. PLoS One.

[56] Jo S, Kim T, Iyer VG, Im W (2008). CHARMM-GUI: a web-based graphical user interface for CHARMM. J Comput Chem.

[57] Abraham MJ (2015). GROMACS: high performance molecular simulations through multi-level parallelism from laptops to supercomputers. SoftwareX.

[58] Lomize MA, Pogozheva ID, Joo H, Mosberg HI, Lomize AL (2012). OPM database and PPM web server: resources for positioning of proteins in membranes. Nucleic Acids Res.

[59] MacKerell AD (1998). All-atom empirical potential for molecular modeling and dynamics studies of proteins. J Phys Chem B.

[60] MacKerell AD (2004). Improved treatment of the protein backbone in empirical force fields. J Am Chem Soc.

[61] Klauda JB (2010). Update of the CHARMM all-atom additive force field for lipids: validation on six lipid types. J Phys Chem B.

[62] Venable RM et al (2014). CHARMM all-atom additive force field for sphingomyelin: elucidation of hydrogen bonding and of positive curvature. Biophys J.

[63] Humphrey W (1996). VMD: visual molecular dynamics. J Mol Graph.

