# Supplementary material for: Mandibulofacial dysostosis with alopecia results from ETAR gain-of-function mutations via allosteric effects on ligand binding
Source: J Clin Invest. 2023 Feb 15;133(4):e151536. doi: 10.1172/JCI151536 (PMC9927936; doi:10.1172/JCI151536)
Supplement: Supplemental data [file jci-133-151536-s133.pdf]

Supplemental materials for

Mandibulofacial dysostosis with alopecia results from gain-of-ET_AR function via allosteric effects on ligand binding

Yukiko Kurihara[1*], Toru Ekimoto[2], Christopher T. Gordon[3], Yasunobu Uchijima[1], Ryo Sugiyama[1], Taro Kitazawa[1], Akiyasu Iwase[1], Risa Kotani[1,4], Rieko Asai[1], Véronique Pingault[3,5], Mitsunori Ikeguchi[2,6], Jeanne Amiel[3,5], Hiroki Kurihara[1]

[1]Department of Physiological Chemistry and Metabolism, Graduate School of Medicine, The University of Tokyo, Tokyo,113-0033, Japan.
[2]Graduate School of Medical Life Science, Yokohama City University, Yokohama, 230-0045, Japan
[3]INSERM UMR 1163, Institut Imagine and Université Paris-Cité, Paris 75015, France
[4]Department of Medical Science, Graduate School of Medicine, University of Hiroshima, Hiroshima, 734-8551, Japan.
[5]Service de Médecine Génomique des Maladies Rares, Hôpital Necker-Enfants Malades, AP-HP, Paris, 75015, France
[6]Center for Computational Science, RIKEN, Yokohama, 230-0045, Japan

*Address correspondence to: Yukiko Kurihara, The University of Tokyo, 7-3-1 Hongo, Bunkyo-ku, Tokyo,113-0033, Japan. Phone:+81-3-5841-3498; Email: yuki-tky@umin.net

Present address:
Taro Kitazawa; Aarhus University, MBG, Universtetbysen 81, 8000 Aarhus C, Denmark
Rieko Asai; Cardiovascular Research Institute, UCSF, California, USA
Ryo Sugiyama; Department of Pediatrics, Graduate School of Medicine, The University of Tokyo, Tokyo,113-0033, Japan
Risa Kotani; Department of Neurology, Graduate School of Medicine, The University of Tokyo, Tokyo,113-0033, Japan

Supplemental Methods

Skeletal staining

Skeletal staining of mouse embryos with alizarin red and alcian blue was performed according to a previously described protocol with slight modifications(1).

In situ hybridization

Whole-mount in situ hybridization of E9.5 embryos was performed as described previously(2). Probes were generated using a DIG RNA labeling kit (Roche) from a plasmid containing the mouse Dlx5 or Dlx6 sequence as a template (3).

Quantitative real-time RT-PCR

Mandibular and maxillary parts of the first pharyngeal arch were isolated from E9.5 embryos. Total RNA was extracted from a pool of each of these tissues from 7 to 14 littermates, from each of four pregnant mice, using the RNeasy® Mini Kit according to the manufacturer's protocol. cDNA was transcribed from 3μg of total RNA with ReverTra Ace® (TOYOBO) and mRNA levels were quantified by qRT-PCR using a Light Cycler® FastStart DNA Master SYBR Green kit. The second-derivative maximum method was adopted to determine the crossing points automatically for individual samples. Glyceraldehyde-3-phosphate dehydrogenase (*Gapdh*) was used as an internal control. The results were expressed as a fold change of the maxillary value relative to the mandibular value for each litter of embryos. The primers are shown in Supplementary Table 4.

Measurement of ERK phosphorylation

HeLa cells grown in 12-well plates were transfected with pCEFL expression plasmids coding mouse WT, Y129F, E303K or other E303 mutant ET_AR by Lipofectamine® LTX with Plus™ Reagent (Thermo Fisher Scientific). The equivalent expression level of each ET_AR was confirmed by qRT-PCR (data not shown). After starvation with 0.1% FCS in DMEM for 7 hours, ET1 or ET3 was added to the cells between at concentrations from 10^{-12} to 10^{-7} M, and the reactions were stopped by liquid nitrogen just after 15min. The extracted proteins were applied to SDS-PAGE, and transferred to PVDF membranes (Immobilon, MERCK). Primary antibody was anti-

total p44/42 MAPK (Erk1/2) antibody (Cell Signaling Technology Cat# 9102, RRID:AB_330744) and secondary antibody was anti-rabbit IgG conjugated with horseradish peroxidase (DAKO Cat# P0399, RRID:AB_2617141). Total Erk1/2 proteins were detected by enhanced chemiluminescence (ECL Prime, Amersham) with ImageQuant LAS4000 mini (GE Healthcare). After stripping anti-total MAPK (Erk1/2) antibody, membranes were hybridized with anti-phospho-p44/42 MAPK (Erk1/2) (Thr202/Tyr204) antibody (Cell Signaling Technology Cat# 9106, RRID:AB_331768), and the secondary antibody was anti-mouse IgG conjugated with horseradish peroxidase (DAKO Cat# P0447, RRID:AB_2617137). We analyzed the density of each band by ImageJ and calculated the ratio of phosphorylated to total Erk1/2, and the value at ET concentration of 10^{-7}M was set to 100%. EC_{50} values were calculated from least squares nonlinear regression using GraphPad Prism 8 software. HeLa (ATCC, CCL-2) and HEK293 (ATCC, CRL-1573) cells were obtained from ATTC and grown. No additional specific authentication was performed.

Measurement of intracellular Ca^{2+} levels

HeLa cells were spread onto black-wall clear-bottom 96-well plates at a density of $2x10^4$ cells/well and transiently transfected with expression plasmids encoding WT, Y129F, E303K or other E303 mutant ET_AR using Lipofectamine LTX. In addition, cells transfected with Au5 tag-only plasmid served as a control. Cells were allowed to grow for 12 h, then serum-deprived for 1h for measurement of basal activity, or in 0.1% FCS in DMEM for a further 6h for the ligand response curve. To measure intracellular Ca^{2+} levels, we used Screen Quest™ Fluo-8 No Wash Calcium Assay kit (AAT Bioquest, Cat# 36315) according to the manufacturer's protocol. Briefly, cells were incubated in serum-free medium with the same amount of Fluo-8 NW dye-loading solution for 1 hour at room temperature. Then, different concentrations of ligands were added and intracellular Ca^{2+} levels were monitored for 15 min at 15 sec intervals by fluorescence intensity at Ex/Em=485/535nm using TECAN Infinite Pro 200. The maximum peak intensity during measurement was obtained for each well. EC_{50} values were calculated from least squares nonlinear regression using GraphPad Prism 8 software.

Because basal activity compares slight differences between different proteins unlike the agonist-activation system for calculating EC50, an average of 6 wells measured

for each group simultaneously (that is, 24 wells at a time) was determined and the value of Au5 negative control-transfected cells was subtracted as a background. The data were expressed as a fold change relative to the WT value. This process was repeated 11 times independently.

IP_1 assay

HeLa cells in 24-well plates were transfected with 1.5μg of expression plasmids encoding ET_AR-WT, Y129F, E303K, or Au5-tag (control) using Lipofectamine LTX®. They were cultivated for 18 h in growth medium then in serum-deprived medium for 1 hour. For evaluating the response to ET3, different concentrations of ET3 prepared in Stimulation Buffer containing 50mM LiCl to prevent IP_1 degradation were added for 1h, and 14μl of lysate was transferred to wells of a HTRF 96-well low volume plate containing 3 μl of d2 conjugate-IP_1 and 3 μl of europium cryptate-labelled anti-IP_1 antibody dissolved in 1% Triton lysis buffer. After a further incubation of 1 h at room temperature, time-resolved fluorescence was measured at Ex/Em=337/620nm for d2 and Ex/Em=337/665nm for europium cryptate with the PHERAstar multimode reader. A standard curve was prepared for each experiment exactly according to the manufacturer's instruction, and IP_1 concentrations were calculated from the standard curve. EC_{50} values were calculated from least squares nonlinear regression using GraphPad Prism 8 software.

For basal activity, two-hour starved lysate was transferred to a HTRF 96-well plate in an amount of 1, 1/2, or 1/4 volume, in four replicate wells for each volume, with each well containing d2-IP_1 and anti-IP_1 antibody. The fluorescence was measured after incubation for 1h, and IP_1 concentrations were calculated from a standard curve. Using the mean values of the four replicate wells containing relative cell numbers of 0.25, 0.5 or 1, a simple linear regression line was drawn, and the value of the slope was obtained. These slope data were expressed as a fold change relative to the WT value. This process was repeated six times independently.

Ligand binding assay

The ligand binding assay was performed as described previously(4). Briefly, HEK293 cells were transfected with pCEFL expression vectors coding mouse ET_AR-WT, Y129F or E303K ET_AR mutants by Effectene transfection reagent (Qiagen) in 100 mm dishes. After 48 hours, cells were harvested, and the crude membranes were prepared. Equivalent mRNA expression levels for WT and mutant ET_ARs were confirmed by qRT-PCR (data not shown). In the competitive binding assays, HEK293 membranes containing 1~2 μg proteins were incubated with 0.03–0.04 nM ^{125}I-labelled Endothelin-1 (2,200 Ci mmol−1, PerkinElmer Life Sciences), and eight concentrations of unlabelled ET1 or ET3 (ranging from 1 pM to 1 μM) in 50 μl of 50 mM HEPES-NaOH, pH 7.5, 10 mM $MgCl_2$ (Mg-HEPES) containing 0.1% BSA. Binding reactions were incubated at 37 °C for 1 h, terminated by dilution with ice-cold Mg-HEPES, and filtered onto glass fiber filters in 96-well plates (Multiscreen HTS FB) to separate the unbound ^{125}I-labelled ET1. After three washes with ice-cold Mg-HEPES, the radioactivity captured by the filters was counted using a γ counter. The reactions were repeated two times. The results were analyzed by nonlinear regression, using the GraphPad Prism 7 software. In the saturation binding assay, membranes containing 0.5~0.7 μg proteins were incubated with eight different concentrations of ^{125}I-labelled ET1, ranging from 2 pM to 150 pM in 50 μl of Mg-HEPES containing 0.1 % BSA, at 37 °C for 2h. The non-specific binding of the ^{125}I-labelled ET1 was measured by including 100 nM ET1 in the same reaction. The membranes were isolated from the unbound ^{125}I-labelled ET1 and washed, and the amount of receptor–bound ^{125}I-labelled ET1 was measured as described above. Saturation binding isotherms of WT and mutant receptors showed comparable Kd and Bmax values as follows:

	Kd (pM)	Bmax (pM)
WT	30.5 ± 3.9	17.8 ± 0.7
Y129F	28.3 ± 4.5	18.2 ± 0.9
E303K	33.1 ± 6.8	23.4 ± 1.5

The apparent dissociation constants of ET1 were determined by fitting to a one-site binding equation, using the GraphPad Prism 8 software.

ET_AR reconstitution in HDL particle

WT and mutant ET_AR constructs containing an amino-terminal Flag epitope tag followed by a hexa-histidine tag were expressed in Sf9 insect cells using the Bac-to-Bac baculovirus system. Solubilization of Sf9 cell-expressed ET_ARs from 300 ml culture in 1% digitonin yielded approximately 1 nmol ^{125}I-labelled ET1-binding activity. Flag-ET_ARs were purified using Flag-M2 resin and subsequent TALON Metal affinity resin in 0.1% digitonin, 20 mM Tris-HCl (pH 7.5), 0.3 M NaCl and 100 mM imidazole. Recombinant membrane scaffold protein1 (MSP1) using a MSP1E3D1 construct was expressed in *E. coli* and purified with Ni-NTA affinity resin (Ni-NTA agarose). Flag-ET_ARs were reconstituted into HDL particles according to published methods (5, 6). In brief, Flag-ET_ARs containing 50-100 pmol ligand binding activity in 200 μl volumes were incubated with 60-78 μM MSP1 and 7.5 mM POPC:POPG (3:2 molar ratio) for 1h at 4 °C. Detergent was removed via BioBeads (BioRad) overnight at 4 °C, and receptor-containing nanodiscs were isolated using Flag resin. The ^{125}I-labelled ET1-binding activities in the rHDL particles were quantitated by saturation binding using glass fiber filters described above to align the HDL amount for each receptor. For quantitation of ligand binding activity, we used B_{max} values obtained from saturation binding followed by fitting to a one-site binding equation, using the GraphPad Prism 8 software.

G protein activation assay

WT and mutant ET_AR proteins in the recombinant HDL at 1.8 nM, based on the Bmax values calculated in the saturation bindings were preincubated with Gαq (KeraFAST, purified from insect cells) at 300 nM and $G\beta_1\gamma_2$ subunits (purified from Sf9 cells) at 140 nM at 4 °C for 1 h in 20 mM HEPES-NaOH, pH 8.0, 1 mM EDTA, 100 mM NaCl, 10 mM $MgCl_2$ and 1 mM dithiothreitol, and 1 μM GDP, as described previously(4). The mixtures were divided into 20 μl aliquots and further incubated with or without different concentrations of ET1 or ET3 (ranging from 100 nM to 1 μM) for 5 min at 30 °C. The reactions were then started by adding [^{35}S]GTP-γS at 110 nM and incubated for 5 min at 30 °C. [^{35}S]GTP-γS (1,250 Ci /mmol) was used after dilution with unlabelled GTP-γS to 113.6 Ci /mmol. The reactions were terminated by adding ice-cold stopping buffer containing 100 μM GTP, in 20 mM Tris-HCl, pH 8.0, 25 mM $MgCl_2$ and 100 mM NaCl, and filtered onto cellulose-mixed ester filters in 96-well plates (Multiscreen HTS HA), to isolate the G proteins from the unbound

[^{35}S]GTP-γS. After three washes with ice-cold stopping buffer without GTP, the radioactivity of the bound [^{35}S]GTP-γS was measured, using a liquid scintillation counter. The amount of [^{35}S]GTP-γS bound without agonist addition was subtracted as a background from each sample stimulated with various concentrations of agonist. The assays were repeated two to three times. The data were analyzed using the GraphPad Prism 8 software.

System building for molecular dynamics simulations

In the homology modeling using MODELLER, the structure deposited in the PDB-REDO databank (7) was employed as a template. The input sequence alignment was built using T-COFFEE. In the construction of the complex system using CHARMM-GUI, detailed procedure was as follows. The input structures were the model structure of ET_AR and 16 crystal water molecules near ET_AR. The missing hydrogen atoms were inserted by CHARMM-GUI. The protonation states of histidine were determined by pK_a using the program H++(8-10), and the N_δ atom of histidine was protonated. The N- and C-termini were set to NH_3^+ and COO^-, respectively. The orientation of ET_AR relative to the lipid bilayer was set to that of the ET_BR crystal structure deposited in OPM database (11)(ID: 5GLI): The model ET_AR structure was aligned to the ET_BR structure by least-square fitting using Cα atoms. The MD unit cell was set to a rectangular cell. In the center of the cell, the model ET_AR structure was embedded in the lipid bilayer in the x-y plane. The lipid bilayer was a 55.3 Å × 55.3 Å POPC bilayer, in which the number of POPC molecules at the upper and lower leaflets was 26 and 28, respectively. Along the z-axis of the unit cell, water molecules (TIP3P water model (12)) were added. The water thickness was set to 17.5 Å. Counterions (Na^+ ion) and 150 mM NaCl were included. The system size for the ET_AR-WT was 55.3 × 55.3 × 110.0 Å^3. In the mutant ET_AR, Na-bound ET_AR, or ET-bound ET_AR, their complex systems were constructed using the same procedure described above.

Procedures of Molecular Dynamics Simulation

According to the default settings of the CHARMM-GUI input generator (13), an energy minimization (steepest descent method) and six simulations for equilibration,

hereafter denoted as (i)-(vi), were performed before production runs. All simulations were performed using GROMACS with periodic boundary conditions. The Electrostatic interactions were handled by the smooth particle mesh Ewald method (14). The van der Waals interaction was smoothly truncated using the switching function within a range of 10-12 Å. Bond lengths containing hydrogen atoms were constrained by the P-LINKS algorithm (15). The canonical (NVT) ensemble was adopted in the (i) and (ii) runs, and the isothermal-isobaric (NPT) ensemble was adopted in the remaining (iii) to (vi) runs. The thermostat was the weak-coupling scheme of Berendsen (16). The barostat used in the (iii) to (vi) runs was the semi-isotropic Berendsen algorithm (16). The time step was set to 1 fs in the (i) to (iii) runs, and 2 fs in the (iv) to (vi) runs. The simulation lengths were 25 ps for the (i) to (iii) runs, and 100 ps for the (iv) to (vi) runs.

In the energy minimization stage and in equilibration runs, structural constraints were imposed on the protein and the POPC bilayer. The position harmonic constraints were imposed on backbone and sidechain heavy atoms of the protein. During the equilibration runs, the strength of the force constant was gradually reduced from 4000 to 50 ($kJ \cdot mol^{-1} \cdot nm^{-2}$) for the backbone, and 2000 to 0 ($kJ \cdot mol^{-1} \cdot nm^{-2}$) for the sidechain. In POPC molecules, the position harmonic constraints were imposed on the z-axis of the phosphorus atom, and the force constant was gradually reduced from 1000 to 0 ($kJ \cdot mol^{-1} \cdot nm^{-2}$). In addition, two dihedral angles in POPC molecules were constrained: One was the dihedral angle corresponding to the bifurcation point of the sn-1, -2, and -3 positions around the carbon atom at the sn-2 position and was determined as the carbon atom at the sn-2 position, two adjacent carbon atoms at the sn-1 and -3 positions, and one adjacent oxygen atom at the sn-2 position. The angle was restrained to 120 degrees. The other was the dihedral angle corresponding to the double bond between carbon atoms in the oleic acid part at the sn-2 position, and the angle was restrained in the cis conformation. The force constants of the two dihedral angles were gradually reduced from 100 to 0 ($kJ \cdot mol^{-1} \cdot rad^{-2}$).

In the production run, the time step was set to 2 fs. The thermostat was the Nosé-Hoover scheme (17, 18). The barostat was the semi-isotropic Parrinello-Rahman approach (19, 20).

Modeling of Na-bound ET_AR

To compare solution structures of ET_AR with or without a Na^+ ion in the Na/water pocket, the Na^+ ion in the pocket was modeled. The crystal structure of the human A_{2A} adenosine receptor ($A_{2A}AR$, PDBID: 4EIY) was aligned to the ET_AR model structure using $C_α$ and $C_β$ atoms of $D52^{2.50}$ and $S91^{3.39}$ for $A_{2A}AR$, and $D126^{2.50}$ and $T172^{3.39}$ for ET_AR. From the aligned $A_{2A}AR$ structure, one Na^+ ion near $D52^{2.50}$ and crystal water molecules located within 12 Å around the Na^+ ion were extracted. The extracted ion and water molecules in the Na/water pocket were transplanted to the ET_AR model structure, and water molecules that clashed with side chains of ET_AR were erased.

Trajectory analyses

Using snapshots, hydrogen bond, principal component analysis (PCA), and the number of water molecules in the inner part of ET_AR were analyzed. The hydrogen bonds were detected by the Hydrogen Bonds module implemented in VMD. The donor-acceptor distance and the angle cutoff were set to 3.0 Å and 20 degrees, respectively. To examine the structural distributions sampled by the three 1 μs trajectories for each of the WT, E303K mutant and Y129F mutant of apo-ETAR, PCA was performed. Using the nine 1 μs trajectories (9000 snapshots in total), the principal axes were determined, and the reference structure of the PCA was an averaged structure of the extracellular region of the helices 2, 6, and 7 (Cα atoms in residues 129-142, 317-331, and 343-362, respectively). The trajectories of ET3-ET_AR or Na-ET_AR were projected on the PC axes determined by apo-ETAR. The number of water molecules was determined by counting the number of oxygen atoms present in the rectangular cells placed inside ET_AR. Three rectangular cells were placed so that the long side of each was perpendicular to the transmembrane helices (Figure 6A), and the size of one cell was 17 × 17 × 8 $Å^3$. The three cells were comprised of the G-protein binding area (area1), the D1262.50 area (area2) and the Y1292.53 area (area3).

Supplemental References

1. McLeod MJ. Differential staining of cartilage and bone in whole mouse fetuses by alcian blue and alizarin red S. *Teratology* 1980;22(3):299–301.

2. Wilkinson DG. *In situ hybridization : a practical approach*. Oxford University Press; 1998:

3. Tsai SQ et al. Endothelin-1 regulates the dorsoventral branchial arch patterning in mice. *Nature* 2018;558(7711):1–14.

4. Shihoya W et al. Activation mechanism of endothelin ET B receptor by endothelin-1. *Nature* 2016;537(7620):363–368.

5. Vélez-Ruiz GA, Sunahara RK. Reconstitution of G protein-coupled receptors into a model bilayer system: reconstituted high-density lipoprotein particles. *Methods Mol. Biol.* 2011;167–182.

6. Whorton MR et al. A monomeric G protein-coupled receptor isolated in a high-density lipoprotein particle efficiently activates its G protein. *Proc. Natl. Acad. Sci.* 2007;104(18):7682–7687.

7. Joosten RP, Long F, Murshudov GN, Perrakis A. The PDB_REDO server for macromolecular structure model optimization. *IUVrJ.* 2014;1:213–220.

8. Gordon JC, Myers JB, Folta T, Shoja V, Heath LS, Onufriev A. H++: a server for estimating pKas and adding missing hydrogens to macromolecules. *Nucleic. Acids Res.* 2005;33:W368–71.

9. Myers J, Grothaus G, Narayanan S, Onufriev A. A simple clustering algorithm can be accurate enough for use in calculations of pKs in macromolecules. *Proteins* 2006;63:928–938.

10. Anandakrishnan R, Aguilar B, Onufriev A. H++ 3.0: Automating pK prediction and the preparation of biomolecular structures for atomistic molecular modeling and simulation. *Nucleic. Acids Res.* 2012;40:W537–541.

11. Lomize MA, Pogozheva ID, Joo H, Mosberg HI, Limize AL. OPM database and PPM web server: resources for positioning of proteins in membranes. *Nucleic. Acids Res.* 2012;40:D370–376.

12. Jorgensen LW, Chandrasekhar J, Madura DJ, Impey WR, Klein LM. Comparison of simple potential functions for simulating liquid water. *J. Chem. Phys.* 1983;79:926–935.

13. Lee J et al. CHARMM-GUI input generator for NAMD, GROMACS, AMBER, OpenMM, and CHARMM/OpenMM simulations using the CHARMM36 additive force field. *J. Chem. Theory Comput.* 2016;12(1):405–413.

14. Essmann U, Perera L, Berkowitz LM, Darden T, Lee H, Pedersen GL. A smooth particle mesh Ewald method. *J. Chem. Phys.* 1995;103:8577–8592.

15. Hess B. P-LINKS: A parallel linear constraint solver for molecular simulation. *J. Chem. Theory Comput.* 2008;4:116–122.

16. Berendsen HJC, Postma JPM, DiNola A, Haak JR. Molecular dynamics with coupling to an external bath. *J. Chem. Phys.* 1984;81:3684–3690.

17. Nosé S. A molecular dynamics method for simulations in the canonical ensemble. *Mol. Phys.* 1984;52:255–268.

18. Hoover WG. Canonical dynamics: equilibrium phase-space distributions. *Phys. Rev. A.* 1985;31:1695–1697.

19. Parrinello M, Rahman A. Polymorphic transitions in single crystals: A new molecular dynamics method. *J. Appl. Phys.* 1981;52:7182–7190.

20. Nosé S, Klein ML. Constant pressure molecular dynamics for molecular systems. *Mol. Phys.* 1983;50:1055–1076.

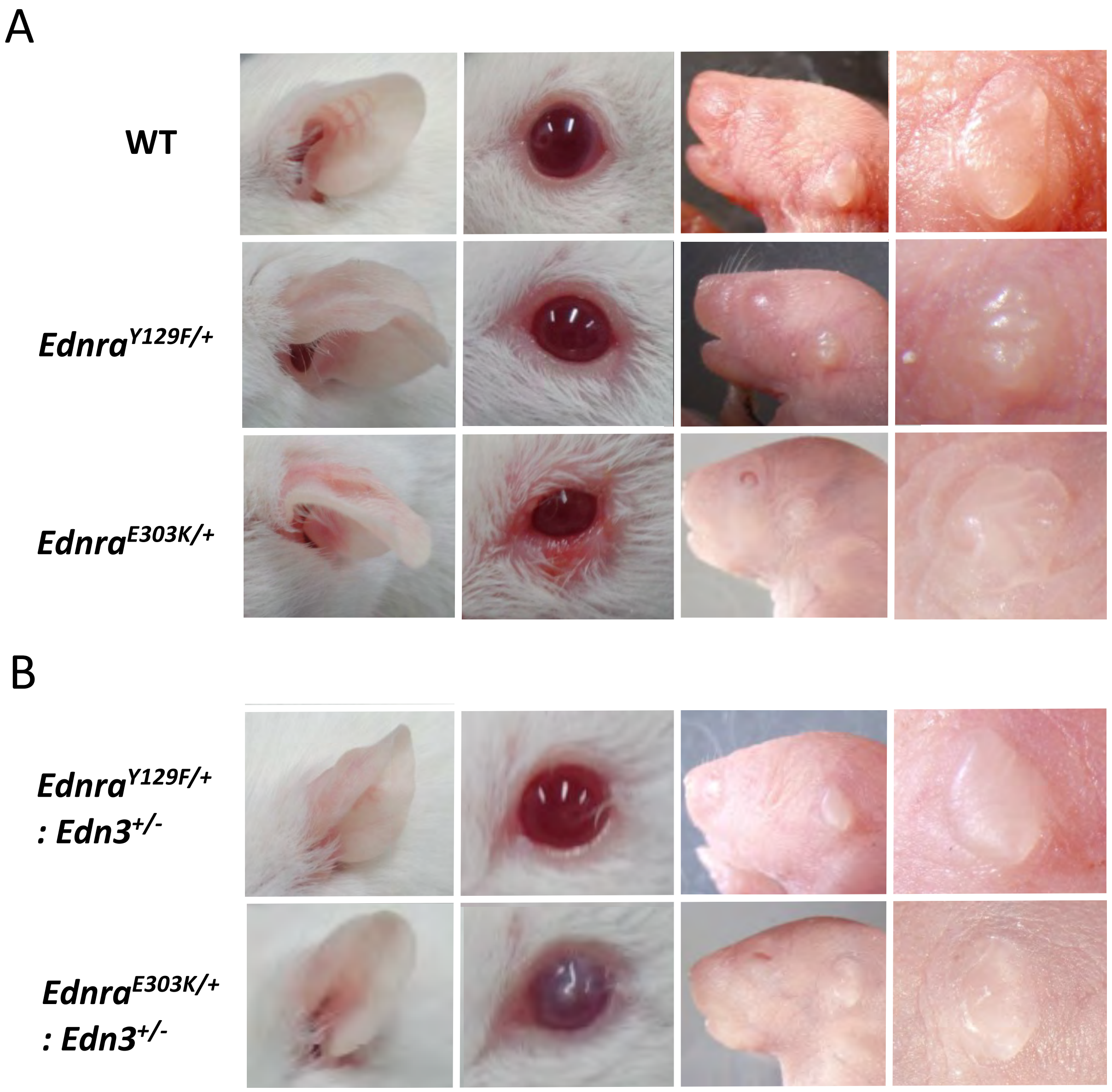

Supplemental Figure 1. The pinnae and eyes of mutant *Ednra* mice are partially rescued by heterozygous *Edn3* deletion. **(A)** The pinnae and eyes of adult (left) and newborn (right). Wild type, *Ednra*$^{Y129F/+}$ and *Ednra*$^{E303K/+}$ mice are shown. **(B)** Heterozygous loss of *Edn3* alleviates the pinna and eye phenotypes of adult (left) and newborn (right) *Ednra*$^{Y129F/+}$ and *Ednra*$^{E303K/+}$ mice.

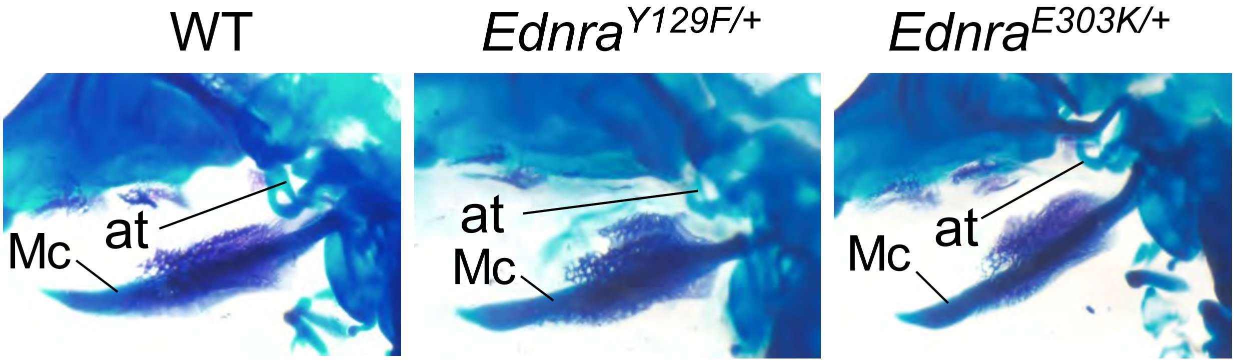

Supplemental Figure 2. Comparison of the chondrocranium among E14.5 WT, *Ednra*$^{Y129F/+}$ and *Ednra*$^{E303K/+}$ mice. There is neither an ectopic Meckel's cartilage (Mc) nor a deformed ala temporalis in the mutants. (WT: n=14, *Ednra*$^{Y129F/+}$; n=3, *Ednra*$^{E303K/+}$; n=5)

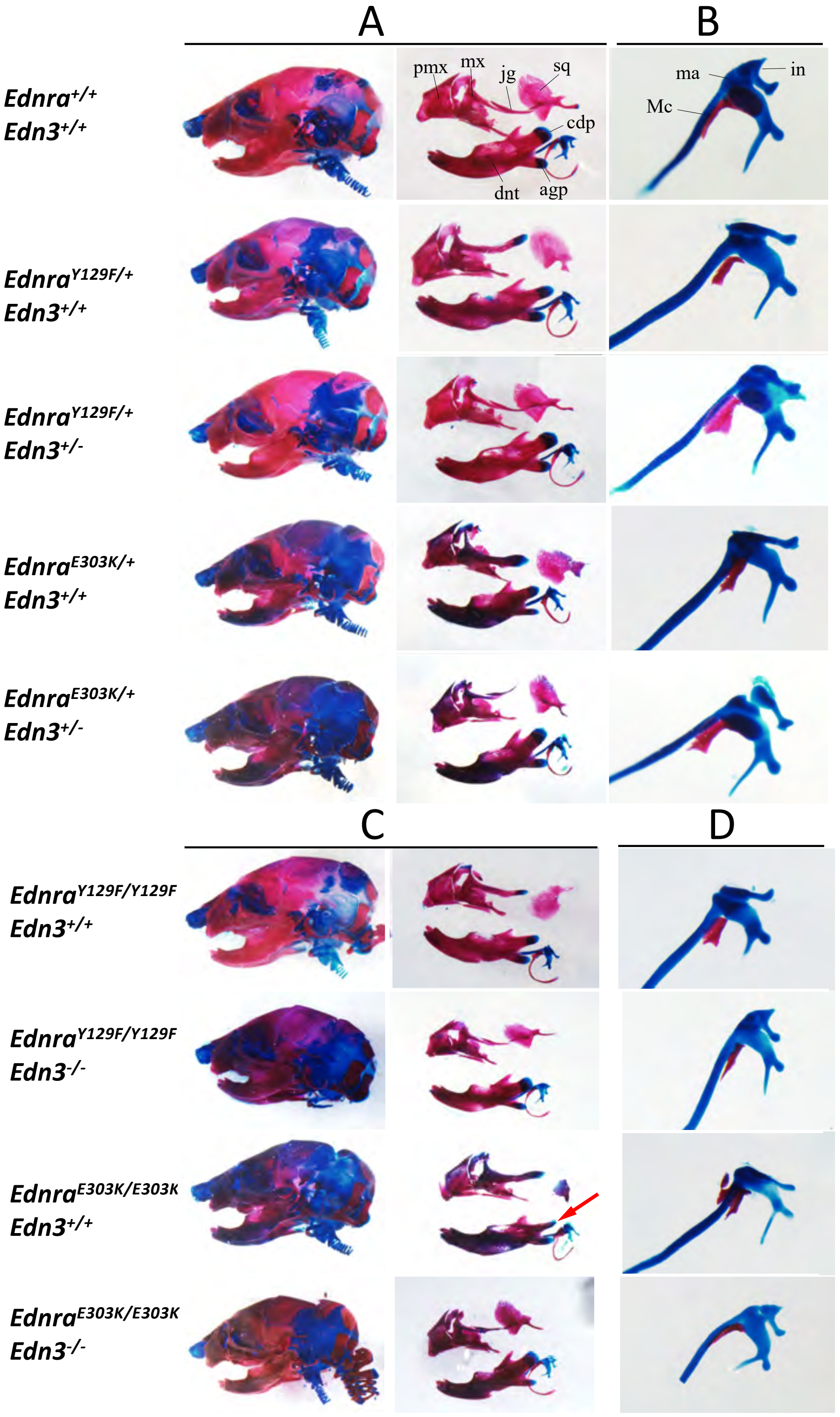

Supplemental Figure 3. Craniofacial skeletons. Craniofacial skeletons (A,C) and PA1-derived auditory ossicles (B,D) of WT mice and heterozygous (A,B) and homozygous (C,D) *Ednra* mutants with different *Edn3* alleles. agp, angular process; cdp, condylar process; dnt, dentary; in, incus; jg, jugal; ma, malleus; Mc, Meckel's cartilage; mx, maxilla; pmx, premaxilla; sq, squamosal. Red arrow in (C) indicates a reduced condylar process.

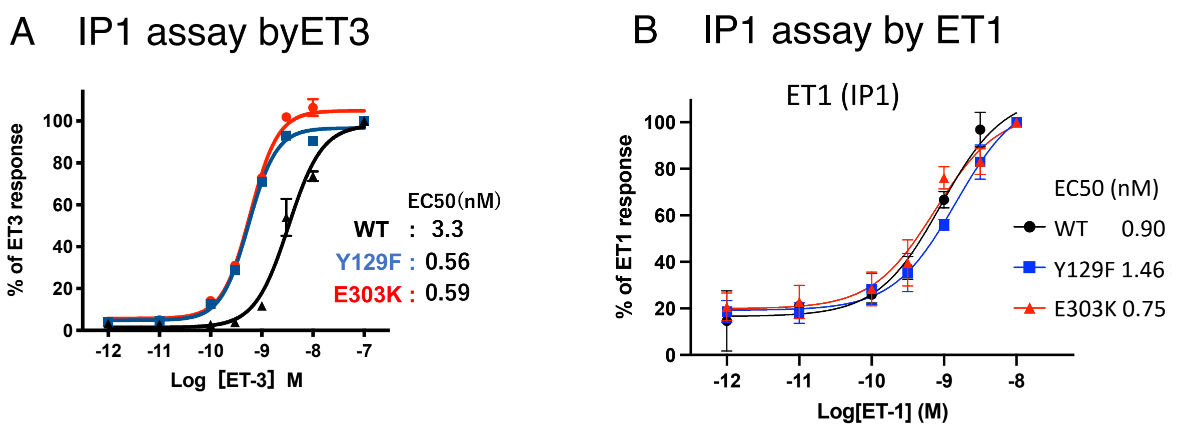

Supplemental Figure 4. IP_1 assay. IP_1 assay upon stimulation by ET3 **(A)** or ET1 **(B)** of cells overexpressing WT or mutant ETAR. Dose-dependent elevation of IP_1 concentration which reflects directly IP_3 concentration. (n=3)

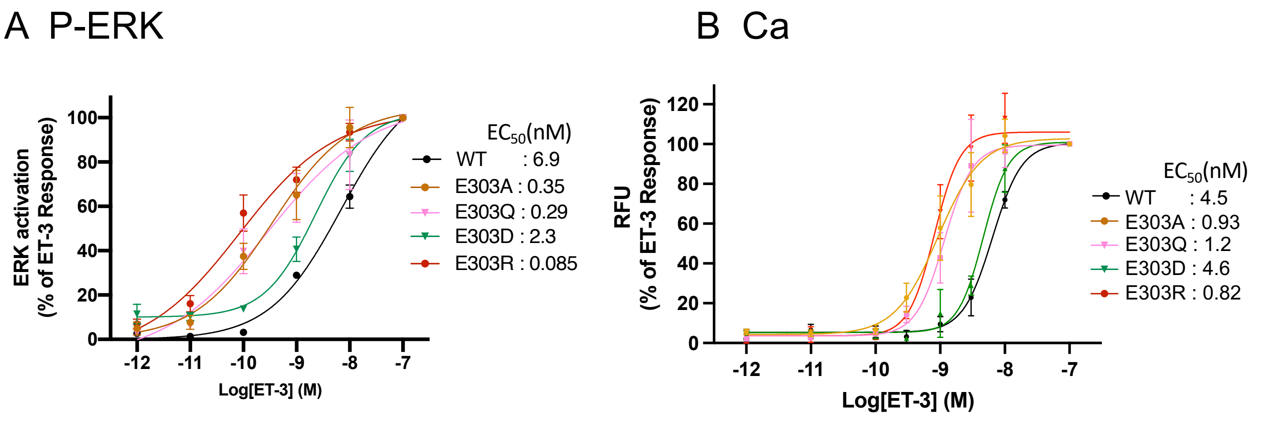

Supplemental Figure 5. Gain-of-function properties of ETAR-E303K is related to the polarity at the position of 303$^{6.32}$. (A) Comparison of ERK phosphorylation between WT and mutant ETARs. Ratios of phosphorylated to total p42/p44 ERK were quantified by Western blot analysis in HeLa cells overexpressing WT ET_AR or E303 mutants with substitution for Ala (E303A), Gln (E303Q), Asp (E303D), or Arg (E303R), upon stimulation by ET3 (n=3). (B) Intracellular calcium mobilization assay in cells overexpressing WT ETAR or substitution mutants showing changes in calcium concentrations in response to ET3 (n=4). RFU, relative fluorescent unit.

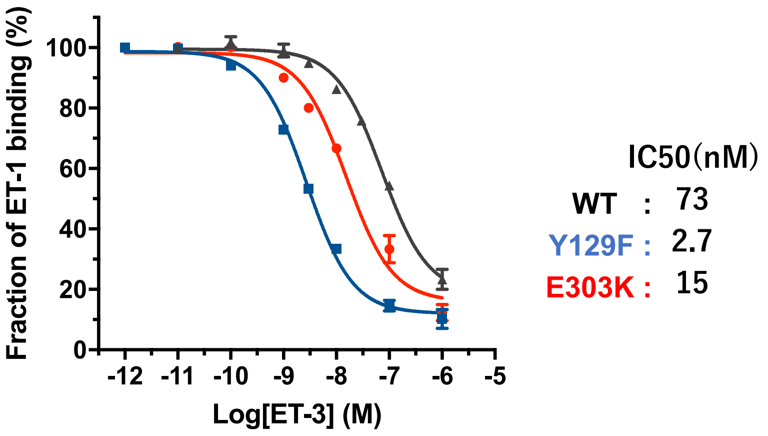

Supplemental Figure 6. Ligand binding on rHDL. Competitive ligand binding assay performed on rHDL particles containing WT or mutant receptors. Each value represents the mean ± SEM from 2-3 independent experiments performed in duplicate.

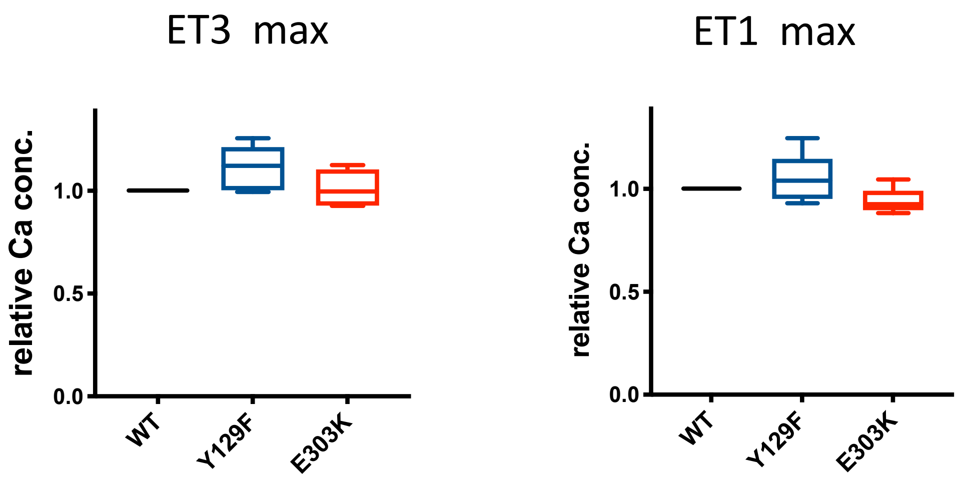

Supplemental Figure 7. Maximal calcium responses. Measurement of intracellular calcium concentrations upon stimulation by 10^{-7}M of ET3 or ET1. Each value of ET_AR-WT, -Y129F, and -E303K was measured simultaneously, and is normalized to the value of WT ET_AR-expressing cells (n=6 each). Steel-Dwass's multiple comparison test is applied for comparison. No significant differences are detected between WT and mutants.

A

WT

donor	acceptor	occupancy
SER373-Side	**GLU303-Side**	34.33%
LYS374-Side	**GLU303-Side**	19.77%
ARG301-Side	ASN284-Main	14.23%
LYS374-Main	**GLU303-Side**	14.10%
ARG301-Side	MET282-Main	10.07%
ARG301-Side	SER189-Main	6.00%
LYS375-Side	**GLU303-Side**	3.70%
LYS375-Side	GLU296-Side	3.07%
LYS375-Side	GLN300-Side	2.47%
LYS374-Side	GLN300-Side	2.07%
LYS375-Main	**GLU303-Side**	1.47%
ARG301-Side	VAL187-Main	1.03%

Y129F

donor	acceptor	occupancy
SER373-Side	**GLU303-Side**	15.03%
LYS374-Main	**GLU303-Side**	9.90%
LYS374-Side	**GLU303-Side**	7.07%
LYS375-Side	**GLU303-Side**	1.37%
ARG301-Side	ALA186-Main	1.33%

E303K

donor	acceptor	occupancy
GLN300-Side	VAL372-Main	6.87%
LYS374-Side	GLU296-Side	5.17%
**LYS303-Side**	PHE371-Main	1.77%
ARG301-Side	SER189-Main	1.70%
ARG301-Side	ALA186-Main	1.50%
**LYS303-Side**	VAL372-Main	1.50%
ARG301-Side	VAL187-Main	1.17%
ARG301-Side	SER189-Side	1.13%

B

S373E

donor	acceptor	occupancy
THR307-Side	GLU373-Side	15.73%
LYS374-Side	**GLU303-Side**	14.00%
ARG301-Side	SER189-Main	6.43%
ARG301-Side	VAL187-Main	3.57%
ARG302-Side	TYR275-Side	1.00%
ARG301-Side	GLU281-Side	1.00%

C

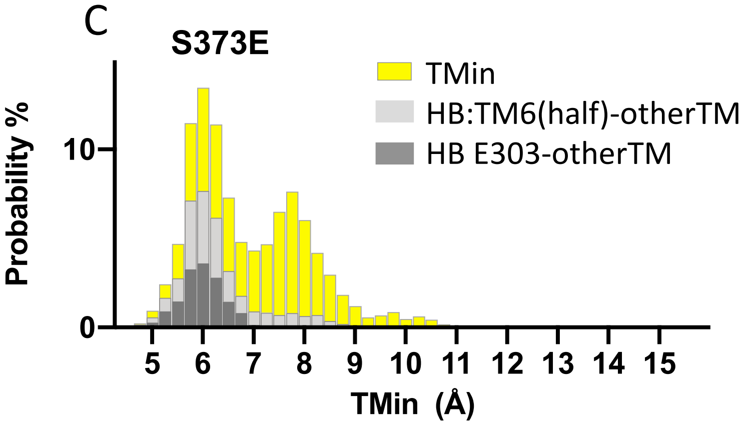

Supplemental Figure 8. The hydrogen bonds between TM6 and other TMs. (A) Lists of hydrogen bonds (HBs) between residues of TM6 (from E296 to C318) and those of other helices in WT and mutant (Y129F, E303K) ET_ARs. Occupancy indicates the number of snapshots displaying each HB per 3000 total shots (%). This list shows HBs with occupancy greater than 1%. HBs involving residue 303 are highlighted in bold. **(B)** Lists of HBs as defined in (A) in ET_AR-S373E. **(C)** Probability distribution of TMin in ET_AR-S373E. HBs formed between the cytoplasmic half of TM6 (from E296 to C318) and other TMs (light gray) and between the 303 residue and other TM residues (dark gray) are superimposed on total TMin distances (yellow).

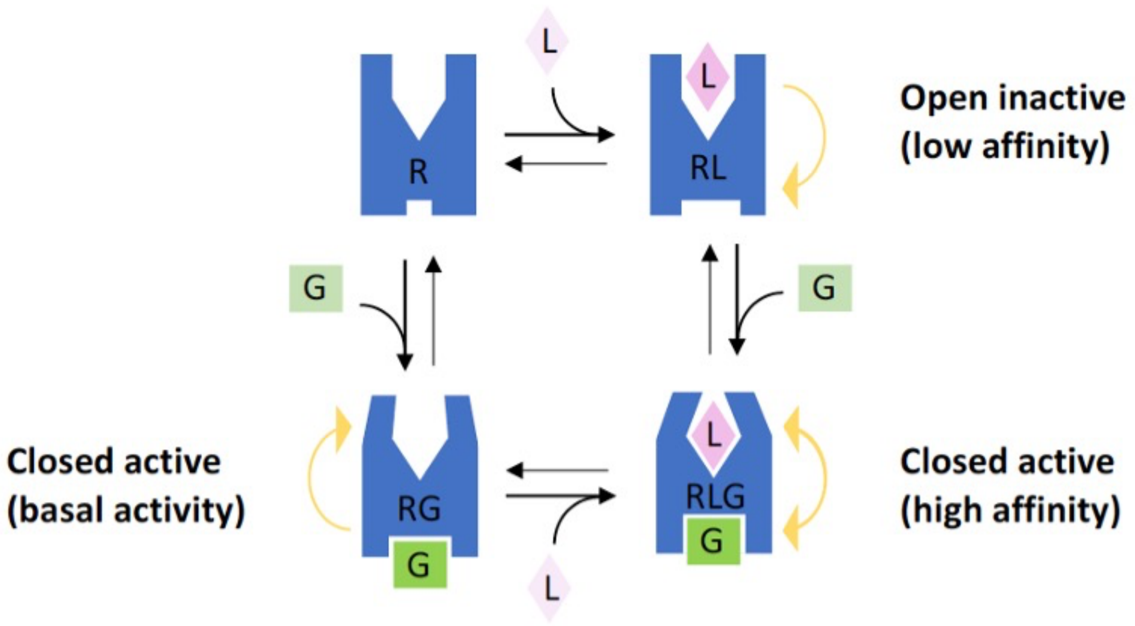

Supplemental Figure 9. Schematic depicting the allosteric effect of G proteins on GPCRs. Upon binding of ligand to the GPCR, the G protein binding site changes its conformation to an open inactive form. Alternatively, G protein bound to receptor without ligand shows basal activity and allosteric conformational change of the ligand binding site to a closed active form. The ternary complex of RLG is a high affinity-closed active form. This figure is modified from previous references (Devree et al., 2016)(Staus et al., 2016a) R; GPCR, G; G protein, L; ligand.

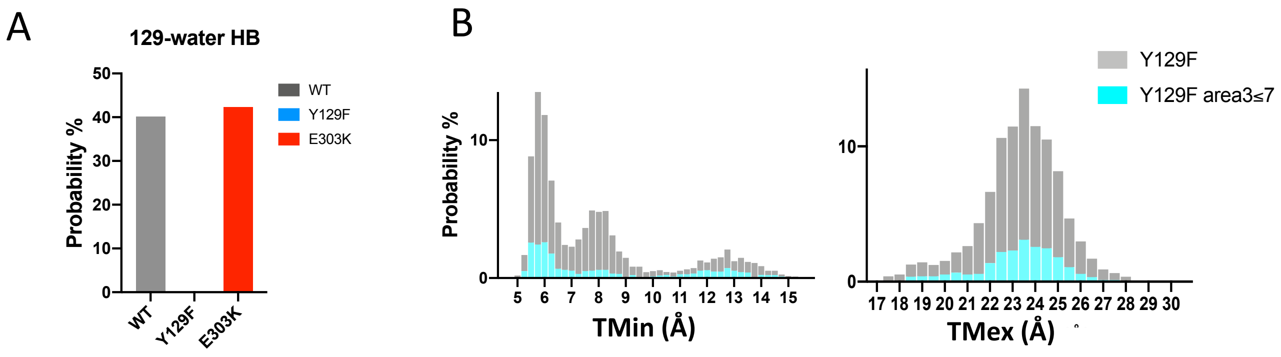

Supplemental Figure 10. The decrease in water molecules around F129 in ETAR-Y129F (A) Probability of hydrogen bonding between the amino acid at position129 and water molecules. **(B)** Distribution of TMin and TMex distance for ET_AR-Y129F (grey) overlaid with structures having an area 3 water molecule count of ≤ 7 (sky blue)

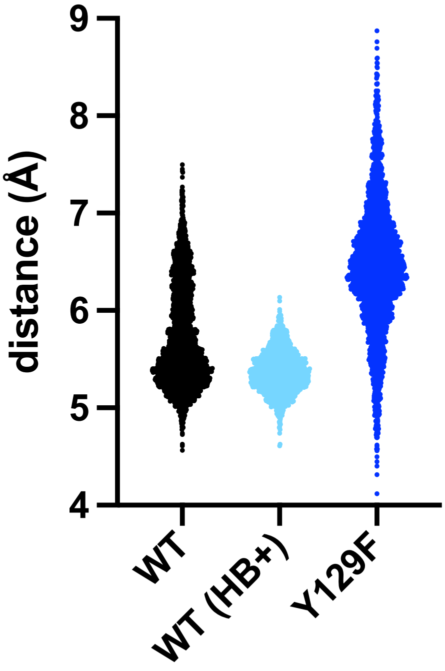

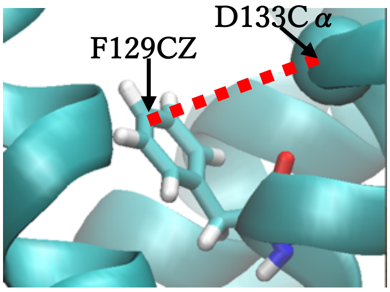

Supplemental Figure 11. Flexible movement of the F129 side chain. The distance between the main chain of D133 (D133Cα atom) as a reference point and the side chain of Y/F129 (Y/F129 CZ atom) are plotted. In WT, the distance as a whole and only when a HB is formed between Y129 and D133 are plotted in black and sky blue, respectively. Note that ETAR-Y129F does not form a HB between F129 and D133.

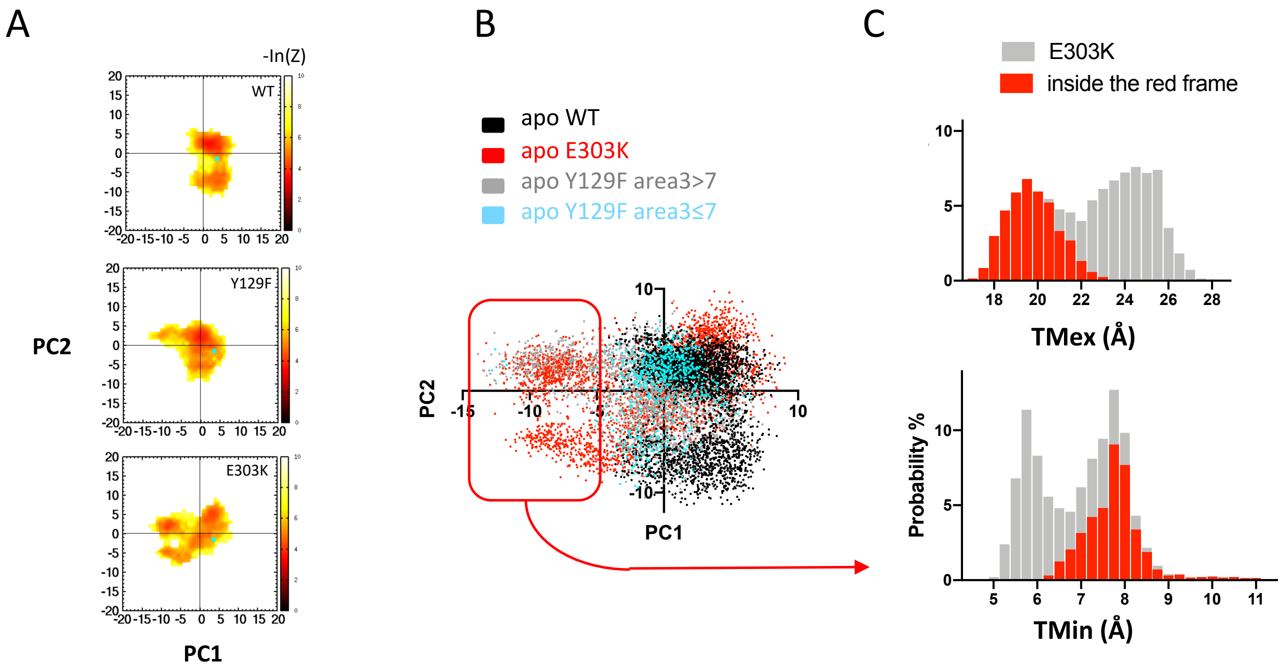

Supplemental Figure 12. Validation of PCA for dynamics analysis. **(A)** The structural distribution of the extracellular half of TM2, 6, and 7 displayed on a principal component surface. The distribution of apo ET_AR-WT, -Y129F, and -E303K are represented as a two-dimensional normalized histogram of -ln(Z) plotted on a PC1-PC2 map with a pixel size of 1Å× 1Å, where Z is the probability of a conformation. The initial structure is represented by a cyan square. The high frequency sections are shown by dark shading, and the low frequency sections are shown by light shading, as indicated on the right axis. **(B)** Comparison of ET_AR structural distributions overlaid on a PC surface. Each snapshot is represented by a dot, and snapshots for apo ET_AR-WT, -Y129F, and -E303K are superimposed. Apo Y129F data were divided into area3>7(light grey) and area3≤7 (sky blue). One of the mutant ET_AR-specific areas is surrounded by a red square (PC1 <-5). **(C)** Probability distribution of TMex and TMin for the mutant ET_AR-specific conformation of apo ET_AR-E303K (red columns). The mutant specific conformation shows wide TMin and narrow TMex.

A

B

WT				Y129F			
donor	acceptor	occupancy		donor	acceptor	occupancy	
LYS166-Side	TRP21-Side	57.98%		LYS166-Side	TRP21-Side	51.95%	
LYS255-Side	TRP21-Side	16.53%		ARG326-Side	ASP18-Side	48.88%	
TYR352-Side	LEU17-Main	12.33%		ARG326-Side	TRP21-Side	33.02%	
GLN165-Side	ILE20-Main	12.03%		LYS255-Side	TRP21-Side	27.72%	
LYS140-Side	ASP18-Main	11.46%		TYR352-Side	LEU17-Main	25.56%	
TYR6-Side	GLN252-Side	6.86%		ARG340-Side	GLU10-Side	24.06%	
				ARG340-Side	ASP8-Side	21.09%	
				TYR13-Side	THR73-Main	16.23%	
				LYS330-Side	THR2-Side	13.30%	
				TRP21-Side	HSD323-Side	8.10%	
				TRP21-Side	ASP256-Side	7.06%	
				LYS140-Side	ASP18-Main	6.06%	
MET65-Main	GLU10-Side	26.29%	N-term.	TYR231-Side	GLU10-Side	29.56%	ECL
TYR231-Side	GLU10-Side	23.93%	ECL	MET65-Main	GLU10-Side	28.92%	N-term.
CYS69-Main	GLU10-Side	23.19%	N-term.	CYS69-Main	GLU10-Side	21.89%	N-term.
MET65-Main	ASP8-Side	17.56%	N-term.	MET65-Main	ASP8-Side	18.39%	N-term.
LYS9-Side	GLU230-Main	14.43%	ECL	THR238-Side	VAL12-Main	11.33%	ECL
LYS9-Side	GLU230-Side	7.86%	ECL	LYS9-Side	GLU230-Main	10.46%	ECL
HSD16-Side	THR238-Side	5.83%	ECL	TRP146-Main	HSD16-Side	8.73%	ECL
				LYS9-Side	GLU230-Side	6.53%	ECL

ET3

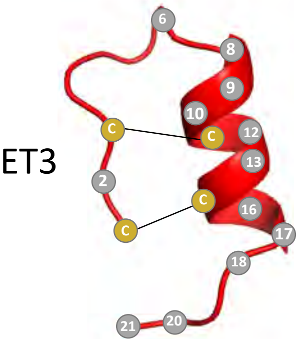

	N-terminal							αHelix										C-terminal			
	1	2	3	4	5	6	7	8	9	10	11	12	13	14	15	16	17	18	19	20	21
ET1	C	S	C	S	S	L	M	D	K	E	C	V	Y	F	C	H	L	D	I	I	W
ET3	C	T	C	F	T	Y	K	D	K	E	C	V	Y	Y	C	H	L	D	I	I	W

Supplemental Figure 13. Amino acid residues involved in HBs between ET3 and ET_AR. **(A)** List of HBs formed between ET3 and ET_AR. HBs with more than 5% probability between residues 1-21 of whole ET3 and residues 65-385 of ET_AR-WT or ET_AR-Y129F are listed. HBs involving amino acid residues outside the cell membrane are shaded with light gray, which show no conspicuous difference between ET_AR-WT and ET_AR-Y129F. **(B)** Structural model of mature ET3, for reference to its interactions with ET_AR listed in (A).

A

donor	acceptor	WT_ET3 occupancy
LYS166-Side	TRP21-Side	57.98%
LYS255-Side	TRP21-Side	16.53%
TYR352-Side	LEU17-Main	12.33%
GLN165-Side	ILE20-Main	12.03%
LYS140-Side	ASP18-Main	11.46%
TYR6-Side	GLN252-Side	6.86%
ARG326-Side	TRP21-Side	4.00%
ARG326-Side	ASP18-Side	2.27%

donor	acceptor	Y129F_ET3 occupancy
LYS166-Side	TRP21-Side	51.95%
ARG326-Side	ASP18-Side	48.88%
ARG326-Side	TRP21-Side	33.02%
LYS255-Side	TRP21-Side	27.72%
TYR352-Side	LEU17-Main	25.56%
ARG340-Side	GLU10-Side	24.06%
ARG340-Side	ASP8-Side	21.09%
LYS330-Side	THR2-Side	13.30%
TRP21-Side	HSD323-Side	8.10%
TRP21-Side	ASP256-Side	7.06%
LYS140-Side	ASP18-Main	6.06%

donor	acceptor	E303K-ET3 occupancy
LYS166-Side	TRP21-Side	53.63%
ARG326-Side	ASP18-Side	30.07%
LYS255-Side	TRP21-Side	29.33%
GLN165-Side	ILE20-Main	17.77%
ARG340-Side	GLU10-Side	17.63%
ARG326-Side	TRP21-Side	8.07%
TYR6-Side	GLN252-Side	5.73%

donor	acceptor	WT+R326Q_ET3 occupancy
ARG340-Side	ASP8-Side	71.10%
LYS166-Side	TRP21-Side	57.87%
GLN165-Side	ILE20-Main	40.37%
LYS255-Side	TRP21-Side	29.40%
ARG340-Side	GLU10-Side	15.73%
LYS140-Side	ASP18-Main	12.57%
TYR352-Side	LEU17-Main	11.07%
LYS140-Side	ASP18-Side	8.43%
ARG340-Main	GLU10-Side	7.23%
PHE4-Main	TYR333-Side	6.27%

donor	acceptor	Y129F+R326Q_ET3 occupancy
LYS166-Side	TRP21-Side	57.85%
ARG340-Side	GLU10-Side	38.82%
LYS255-Side	TRP21-Side	34.26%
TYR352-Side	LEU17-Main	22.96%
ARG340-Side	ASP8-Side	16.33%
GLN165-Side	ILE20-Main	14.30%

donor	acceptor	E303K+R326Q-ET3 occupancy
LYS166-Side	TRP21-Side	58.27%
ARG340-Side	ASP8-Side	54.60%
LYS255-Side	TRP21-Side	31.10%
TYR352-Side	LEU17-Main	29.80%
GLN165-Side	ILE20-Main	28.30%
LYS140-Side	ASP18-Main	17.57%
ARG340-Side	GLU10-Side	9.67%
LYS140-Side	HSD16-Main	9.23%
ILE20-Main	ASN137-Side	5.10%

B

donor	acceptor	WT-ET1 occupancy
LYS166-Side	TRP21-Side	54.97%
ARG340-Side	GLU10-Side	40.17%
ARG340-Side	ASP8-Side	30.30%
ARG326-Side	ASP18-Side	28.93%
ARG326-Side	TRP21-Side	26.27%
LYS255-Side	TRP21-Side	19.40%
GLN165-Side	ILE20-Main	16.07%
TRP21-Side	HSD323-Side	14.40%
LYS140-Side	ASP18-Main	11.13%
TYR352-Side	LEU17-Main	10.93%

donor	acceptor	Y129F_ET1 occupancy
LYS166-Side	TRP21-Side	53.20%
ARG326-Side	ASP18-Side	33.37%
LYS255-Side	TRP21-Side	26.00%
ARG326-Side	TRP21-Side	25.30%
ARG340-Side	ASP8-Side	21.40%
GLN165-Side	ILE20-Main	20.77%
TRP21-Side	HSD323-Side	20.27%
ARG340-Side	GLU10-Side	18.67%
TYR352-Side	LEU17-Main	17.17%
LYS140-Side	ASP18-Main	8.53%
SER2-Side	GLN252-Side	6.50%

donor	acceptor	E303K_ET1 occupancy
ARG326-Side	TRP21-Side	47.33%
ARG340-Side	GLU10-Side	45.30%
LYS166-Side	TRP21-Side	34.83%
ARG340-Side	ASP8-Side	28.77%
TYR352-Side	LEU17-Main	28.63%
TRP21-Side	HSD323-Side	20.27%
LYS140-Side	ASP18-Side	16.13%
LYS140-Side	ASP18-Main	14.73%
GLN165-Side	ILE20-Main	5.50%
TYR13-Side	GLN71-Main	5.33%

donor	acceptor	WT+R326Q_ET1 occupancy
LYS166-Side	TRP21-Side	56.17%
LYS255-Side	TRP21-Side	39.90%
ARG340-Side	GLU10-Side	33.90%
GLN165-Side	ILE20-Main	29.20%
ARG340-Side	ASP8-Side	26.90%
ARG340-Main	GLU10-Side	8.20%
TYR352-Side	LEU17-Main	8.00%
TRP21-Side	HSD323-Side	5.60%
LYS140-Side	ASP18-Main	5.20%

donor	acceptor	Y129F+R326Q_ET1 occupancy
LYS166-Side	TRP21-Side	54.93%
LYS255-Side	TRP21-Side	34.73%
GLN165-Side	ILE20-Main	32.90%
ARG340-Side	GLU10-Side	20.10%
TYR352-Side	LEU17-Main	16.97%
LYS140-Side	ASP18-Main	9.73%
ILE20-Main	ASN137-Side	9.53%
ARG340-Side	ASP8-Side	7.83%
LYS140-Side	ASP18-Side	5.43%

donor	acceptor	E303K+R326Q_ET1 occupancy
LYS166-Side	TRP21-Side	58.47%
ARG340-Side	GLU10-Side	47.63%
LYS255-Side	TRP21-Side	35.70%
GLN165-Side	ILE20-Main	18.70%
LYS329-Side	ASP18-Side	12.80%
TRP21-Side	HSD323-Side	11.80%
TYR352-Side	LEU17-Main	6.67%
GLN71-Side	GLU10-Main	5.87%

Supplemental Figure 14 Hydrogen bonding between ET3(A) or ET1(B) and ET_ARs.

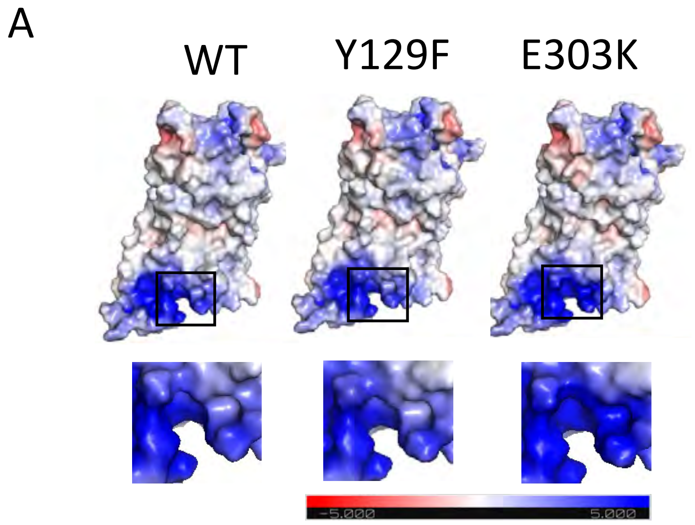

B

H5.	20	21	22	23	24	25	26	
Gs	L	K	Q	Y	E	L	L	-COOH
Gi	L	K	D	C	G	L	F	-COOH
Gt	L	K	D	C	G	L	F	-COOH
Gq	L	K	E	Y	N	L	V	-COOH

Supplemental Figure 15. ETAR-E303K increases the positive charge of the G protein binding pocket, facilitating Gq binding. (A) Surface charge distribution. Whole ET_AR molecules (upper) and the region around the G-protein binding site for each (lower) are displayed (produced by PyMOL). Negative charge; red, positive charge; blue. **(B)** Alignment of the C-terminus of helix 5 (H5) of several G proteins.

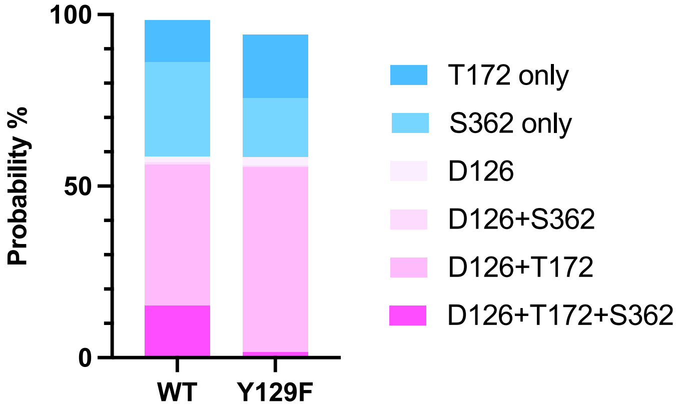

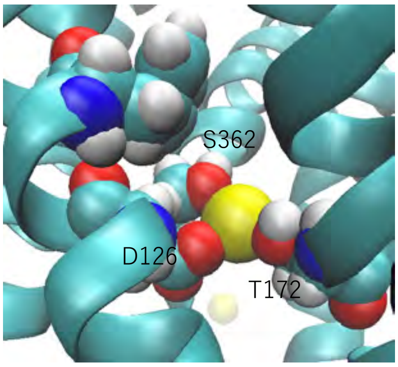

Supplemental Figure16. Sodium ion binding involving TM residues. Amino acid residues forming ionic bonds with sodium ions (left). Representative triple ionic bonds (right). Sodium ion is represented as a yellow sphere.

Clinical history

- 8 year old female
- 3rd child born to non consanguineous parents
- Intrauterine growth retardation
- Born 34 weeks gestation (weight: 1980g, length: 44cm, OFC: 30cm)
- Growth on –2SD for weight and OFC, -1SD for height at 8 years
- Special needs at school, poor concentration span, poor coordination
- Requires hearing aid for conductive deafness

Morphologic findings

- Dysplastic ears
- Unilateral lower eyelid coloboma
- Thin, widely-spaced eyebrows
- Facial asymmetry
- Small open bite
- Squared nasal tip
- Discrete microretrognathia
- Sparse hair and frontal balding
- Diffuse hypopigmentation compared to first degree relatives

Bone findings

- abnormal orientation of the orbital floor
- abnormal temporomandibular articulation on the left side
- hypertrophic, dysplastic and asymmetric malar bones
- hypoplasia of the long process of the incus, dysplastic stapes, head of malleus fused to middle ear wall, reduced volume of middle ear cavity

Genetic analysis

- Array-CGH 100 kb resolution: normal
- Sanger sequencing of *EDNRA* (NM_001957.3): c.907G>A; p.Glu303Lys (mosaic)
- Mandibulofacial dysostosis NGS (Next-generation sequencing) panel: reads showing 8-11% mosaicism of c.907G>A in *EDNRA*

Supplemental Table 1. Detailed patient information.

class A GPCR	active				inactive			
	PDB ID	dist. corresp. to TMin (Å)	Gα	remarks (6.36-7.56)	PDB ID	dist. corresp. to TMin (Å)	binding (antagonist or Na+)	remarks (6.36-7.56)
δ-opioid R					4N6H	**5.5**	sodium ion	M262-L321
μ-opioid R	6DDE	**8.2**	Gi	M281-L339	4DKL	**5.1**	morphinan	M281-L339
adenosine A2A	6GDG	**9.2**	Gs	S234-R291	5VRA	**6.3**	sodium ion	S324-R291
					5UIG	**6.7**	antagonist*	S324-R291
β2AR	3SN6	**11.5**	Gs	T274-R328	5JQH	**6.8**	carazolol, NB60	T1274-R1328
	4LDO	**11.0**	NB80	T1274-R1328	3NYA	**6.8**	alprenolol	T274-R328
5HT	6G79	**7.7**	Go	T315-S372	4IAQ	**5.6**	BRIL	T315-S372
ETBR					5GLI	**6.7**	-	T324-V389
					5XPR	**6.6**	bosentan	T324-V389
					5X93	**6.7**	K-8794	T324-V389
Rhodopsin(human)	6CM0	**8.3**	Gi	M253-M309				
(bovine)	4X1H	**9.0**	Gt	M253-M309	5TE5	**6.7**	mutant retinal	M253-M309

*triazole-carboximidamide (antagonist)-bound

Supplemental Table 2. TMin for some other class A GPCRs. Corresponding to the distance between positions 6.36/Cα-7.56/Cα in crystal structures from GPCRdb (https://gpcrdb.org/structure/) and PDB (https://www.rcsb.org/) measured using PyMOL.

	2.53	6.29	30	31	32	33
5-HT1A receptor	V	R	E	R	K	T
M1 receptor	I	K	E	K	K	A
alpha;1A-adrenoceptor	L	R	E	K	K	A
beta;1-adrenoceptor	M	R	E	Q	K	A
beta;2-adrenoceptor	M	K	E	H	K	A
D1 receptor	V	R	E	T	K	V
H1 receptor	V	R	E	R	K	A
TA1 receptor	L	K	E	R	K	A
AT1 receptor	F	R	N	D	D	I
apelin receptor	F	K	R	R	R	L
BB1 receptor	L	T	R	K	R	L
B1 receptor	F	K	D	S	K	T
CCK1 receptor	L	A	K	K	R	V
C3a receptor	C	-	-	S	K	T
**ETA receptor**	**Y**	Q	R	R	E	V
**ETB receptor**	H	Q	R	R	E	V
FPR1	F	-	S	S	R	P
GAL1 receptor	Y	S	K	K	K	T
ghrelin receptor	I	N	H	K	Q	T
GnRH1 receptor	E	A	R	L	K	T
kisspeptin receptor	F	V	R	A	K	V
MCH1 receptor	F	R	T	K	R	V
MC1 receptor	V	F	G	L	K	G
motilin receptor	I	G	H	R	Q	T
NMU1 receptor	V	G	R	R	Q	V
NPFF1 receptor	V	R	R	A	R	V
NPS receptor	T	A	K	I	K	A
NPBW1 receptor	F	A	K	K	R	V
Y1 receptor	V	E	T	K	R	I
NTS1 receptor	T	A	L	R	H	G
mu; receptor	A	N	L	R	R	I
NOP receptor	V	N	L	R	R	I
OX1 receptor	V	A	R	R	K	T
QRFP receptor	I	K	K	K	R	A
PrRP receptor	M	R	R	R	R	T
PAR1	F	S	K	K	S	R
RXFP1	M	K	E	M	I	L
SST1 receptor	L	S	E	R	K	I
NK1 receptor	M	A	K	R	K	V
TRH1 receptor	V	S	R	K	Q	V
UT receptor	Y	P	G	A	R	A
V1A receptor	V	A	K	I	R	T
OT receptor	V	A	K	I	R	T
FFA1 receptor	L	R	R	K	L	R
BLT1 receptor	V	-	S	R	R	T
CysLT1 receptor	C	S	H	K	K	A
OXE receptor	L	G	P	Q	R	A
LPA1 receptor	A	T	M	M	S	L
S1P1 receptor	A	K	S	L	A	L
CB1 receptor	G	M	D	I	R	L
PAF receptor	F	E	V	K	R	R
DP1 receptor	G	E	E	L	D	H
EP1 receptor	G	H	D	V	E	M
FP receptor	G	H	H	L	E	M
IP receptor	G	D	E	V	D	H
TP receptor	G	D	S	E	V	E
MT1 receptor	V	Q	D	F	R	N
A1 receptor	V	K	E	L	K	I
P2Y1 receptor	Y	P	L	R	R	K
GPBA receptor	T	L	T	W	R	Q
GPER	L	R	R	Q	K	A
HCA1 receptor	L	R	M	K	K	A
oxoglutarate receptor	Y	L	K	Q	K	A
succinate receptor	F	P	L	E	K	P
Rhodopsin	M	A	E	K	E	V
Melanopsin	M	S	E	C	K	M
Opsin-3	V	Y	E	K	K	L
A2A receptor	V	K	E	V	H	A
CONSENSUS	V	R	E	R	K	A

Supplemental Table 3.
Amino acid alignment of human class A GPCRs at the sites corresponding to ET_AR- Y129$^{2.53}$F and around E303$^{6.32}$K mutations. One GPCR is selected as representing each family from GPCRdb (https://gpcrdb.org/alignment/targetselection) except for ETR and βAR.

Related to Figure 3

	WT	Y129F	E303K
A_ET3	13±15	0.092±0.031	0.069±0.036
A_ET1	0.28±0.071	0.23±0.029	0.091±0.024
B_ET3	6.4±0.61	0.70±0.13	0.41±0.051
B_ET1	0.57±0.18	0.59±0.16	0.52±0.091
C_ET3	44±5.7	1.6±0.14	4.5±0.51
C_ET1	0.46±0.053	0.31±0.025	0.43±0.049

(nM)

Related to Figure 7

	WT	Y129F	E303K	WT_R326Q	Y129F_R326Q	E303K_R326Q
D_ET3	6.8±1.6	0.70±0.16	0.46±0.15	-	3.3±0.75	8.7±2.0
D_ET1	0.64±0.12	0.41±0.092	0.30±0.049	2.4±0.51	0.42±0.28	0.24±0.089

Related to Supplemental Figure 4

	WT	Y129F	E303K
A_ET3	3.3±0.51	0.56±0.056	0.59±0.043
B_ET1	0.9±0.23	1.46±0.44	0.75±0.27

Related to Supplemental Figure 5

	WT	Y129F	E303K
ET3	73±7.9	2.7±0.21	15±2.8

Related to Supplemental Figure 6

	WT	E303A	E303Q	E303D	E303R
A_ET3	6.9±2.3	0.35±0.18	0.29±0.29	2.3±0.62	0.085±0.057
B_ET3	6.4±0.25	0.93±0.25	1.2±0.25	4.6±0.81	0.82±0.14

Supplemental Table 4. EC50s±SEM obtained in signaling analysis of ETARs

Gene	Primer name	Sequence (sgRNA-targeting sequence is underlined) (5'-3')	Note
hEDNRA	hEDNRA-S	GGTGTCTGCTACTTCTTGGTAC	
	hEDNRA-AS	GCATGTAGAGTAGCTGGTTTTG	
mEdnra	Y129F-sgRNA-S	TAGGCCTTGGAGACCTTATCTACG	X*: amino acid substitution mutation<br>X**: non-sense mutation<br>X": mutation in the intron
	Y129F-sgRNA-AS	AAACCGTAGATAAGGTCTCCAAGG	
	Y129F-oligo DNA	GCCAGCCTGGCCCTTGGAGAT**CTTATCTT*CGTAGTCATTGACCTCCCCATCAA	
	E303K-sgRNA-894S	TAGGTTGCCACTTCTCGACGCTAA	
	E303K-sgRNA-894AS	AAACTTAGCGTCGAGAAGTGGCAA	
	E303K-oligo DNA	TATCCTTGTTTGCATGAACTA"GT"T"CTTAGCGTCGAA*AAGTGGCAAAGACTGTCTTC	
	Y129F genotype-3728F	CTGCCTAGCAATGGCTCAAT	annealing 60˚C
	Y129F genotype-4013R	AACCTCCTAGGCAGAGCACA	
	E303K genotype-662S	TGCTTTCCTCCTGGCATAGG	annealing 60˚C
	E303K genotype-1018R	TACCTGAGCAGTTCACACCG	
mEdn3	Edn3-sgRNA-918S	TAGGCTGCACAGCCTGAAAGCGCT	
	Edn3-sgRNA-918AS	AAACAGCGCTTTCAGGCTGTGCAG	
	Edn3-sgRNA-1253S	TAGGTTTGTAACTAGGTTCTCAGA	
	Edn3-sgRNA-1253AS	AAACTCTGAACCTAGTTACAAA	
	Edn3 genotype-S1	CTAAAGTCCCACCCCACCAC	annealing 58˚C
	Edn3 genotype-A1	GCTGTGAAGAGACTGTGGCT	

Supplemental Table 5. Primer and oligo DNA sequences
